# Reactional Processes on Osmium–Polymeric Membranes for 5–Nitrobenzimidazole Reduction

**DOI:** 10.3390/membranes11080633

**Published:** 2021-08-17

**Authors:** Aurelia Cristina Nechifor, Alexandru Goran, Vlad-Alexandru Grosu, Andreia Pîrțac, Paul Constantin Albu, Ovidiu Oprea, Alexandra Raluca Grosu, Dumitru Pașcu, Florentina Mihaela Păncescu, Gheorghe Nechifor, Szidonia-Katalin Tanczos, Simona Gabriela Bungău

**Affiliations:** 1 Analytical Chemistry and Environmental Engineering Department, University Politehnica of Bucharest, 1-7 Polizu Street, 011061 Bucharest, Romania; aureliacristinanechifor@gmail.com (A.C.N.); alexandru@santego.ro (A.G.); andreia.pascu@yahoo.ro (A.P.); dd.pascu@yahoo.com (D.P.); florynicorici@yahoo.com (F.M.P.); ghnechifor@gmail.com (G.N.); 2 Department of Electronic Technology and Reliability, Faculty of Electronics, Telecommunications and Information Technology, University Politehnica of Bucharest, Bulevardul Iuliu Maniu, nr. 1-3, 061071 Bucharest, Romania; 3 IFIN Horia Hulubei, Radioisotopes and Radiation Metrology Department (DRMR), 30 Reactorului Street, 023465 Măgurele, Romania; paulalbu@gmail.com; 4 Department of Inorganic Chemistry, Physical Chemistry and Electrochemistry, University Politehnica of Bucharest, 1-7 Polizu Street, 011061 Bucharest, Romania; ovidiu.oprea@upb.ro; 5 Department of Bioengineering, Sapientia Hungarian University of Transylvania, Libertatii Street, 500104 Miercurea-Ciuc, Romania; tczszidonia@yahoo.com; 6 Department of Pharmacy, Faculty of Medicine and Pharmacy, University of Oradea, 410028 Oradea, Romania; sbungau@uoradea.ro

**Keywords:** composite membranes, osmium polymer membrane, nitro derivatives reduction, reactional processes, 5–nitrobenzimidazole, cellulose acetate membranes, polysulfone membranes, polypropylene hollow fiber membranes

## Abstract

Membranes are associated with the efficient processes of separation, concentration and purification, but a very important aspect of them is the realization of a reaction process simultaneously with the separation process. From a practical point of view, chemical reactions have been introduced in most membrane systems: with on-liquid membranes, with inorganic membranes or with polymeric and/or composite membranes. This paper presents the obtaining of polymeric membranes containing metallic osmium obtained in situ. Cellulose acetate (CA), polysulfone (PSf) and polypropylene hollow fiber membranes (PPM) were used as support polymer membranes. The metallic osmium is obtained directly onto the considered membranes using a solution of osmium tetroxide (OsO4), dissolved in tert–butyl alcohol (t–Bu–OH) by reduction with molecular hydrogen. The composite osmium–polymer (Os–P)-obtained membranes were characterized in terms of the morphological and structural points of view: scanning electron microscopy (SEM), high-resolution SEM (HR–SEM), energy-dispersive spectroscopy analysis (EDAX), Fourier Transform Infra-Red (FTIR) spectroscopy, thermogravimetric analysis (TGA) and differential scanning calorimetry (DSC). The process performance was tested for reduction of 5–nitrobenzimidazole to 5–aminobenzimidazole with molecular hydrogen. The paper presents the main aspects of the possible mechanism of transformation of 5–nitrobenzimidazole to 5–aminobenzimidazole with hydrogen gas in the reaction system with osmium–polymer membrane (Os–P).

## 1. Introduction

The membranes and membrane processes represent one of the most viable variants of separation, concentration and purification of the chemical compounds from solutions and complex mixtures [1,2]. The characteristics that recommend the membrane systems for implementation are: high selectivity, flows driven by accessible operational parameters (pressure, temperature and potential difference), simplicity of the modules and their repeatability in large installations, favorable ratio of useful area/installation volume [3,4,5]. These definite advantages of the membrane processes have led to extensive research on various kinds of membranes: liquid, polymeric, inorganic or composite [6,7,8].

At the same time, in order to improve the separation of various chemical species, attempts were made to use their most varied characteristics: dimension, diffusion speed, volatility, surface activity, electric charge, thermal or magnetic field behavior [9,10,11].

However, sometimes there are problems in choosing the membrane so as to ensure a selective and high flow separation, especially if the chemical species to be separated are in very small concentrations or have similar characteristics [12,13].

**Figure 1:**
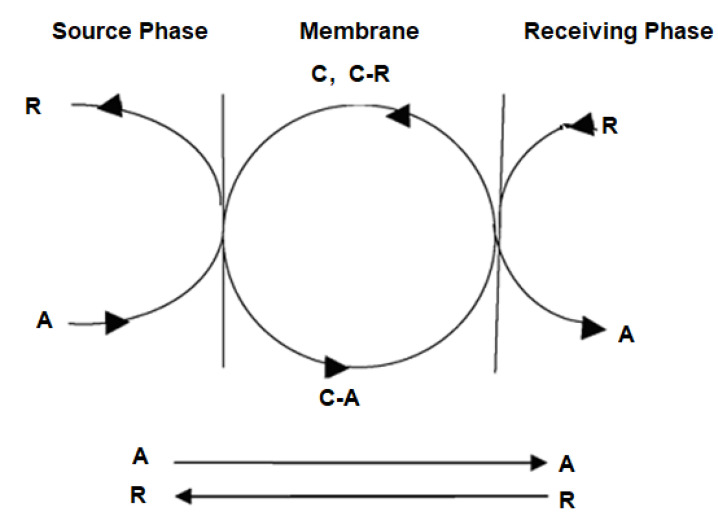
Membrane reaction systems: A—target chemical species; C—carrier; R—reagent.

The separation of chemical species with similar physical–chemical properties has been approached with great insistence and remarkable results in the case of liquid membranes, by introducing a new and versatile variable, the reaction or the chemical interaction of compounds to separate with the membrane components (Figure 1):

- Specific complexants [14,15];
- Ions exchangers [16];
- Surfactants and microemulsions [17,18];
- Ionic liquids [19];
- Adsorbent nanoparticles (polymeric, metallic, oxide) [20,21,22];
- Magnetic nanoparticles [23,24];
- Proteins and enzymes [25,26].

Starting from these results, in the last decade, various membrane reaction systems were addressed, based on polymer and inorganic membranes, but especially on the composite ones, which improve the membrane efficiency in the separation processes, but also add the chemical transformation of the chemical species of interest and developing catalytic or bio-catalytic membrane reactors [27,28,29,30].

The basic element of a membrane reactor is the membrane that ensures both the transformation of the chemical species and their separation [31,32].

When the chemical species for such a process is an organic substance, then the selective transformation and its preferential transfer into the receiving phase are considered [33].

Conventional membrane reactors have ensured esterification, etherification, dehydrogenation, alkylation, synthesis of ammonia or methanol, and in the case of membrane bioreactors, obtaining bioethanol or wastewater treatment [33,34,35].

**Figure 2:**
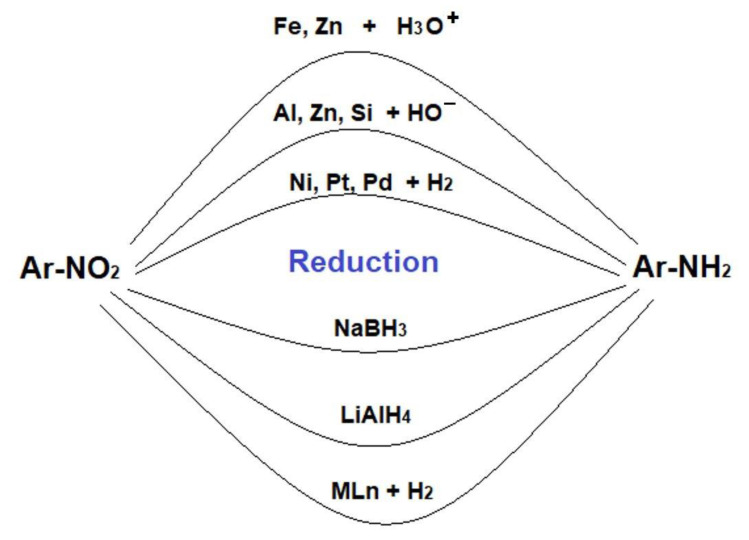
Reduction of aryl-nitro derivatives to anilines.

In particular, the reduction of aryl-nitro derivatives [36,37,38,39,40] represents a continuous challenge, because they are relatively easy to obtain, and their applications are widespread when converted to aryl-amino derivatives (Figure 2).

The transformation of nitro derivatives in homogenous or heterogeneous environment, with hydrogen-generating metals in situ, brings a problem related to the contamination of the organic components with the ions of the metal used. Therefore, a clean hydrogenation using molecular hydrogen by catalysis on composite membranes containing either metal nanoparticles or their complexes is much more promising [41,42,43,44]. Among the metal nanoparticles, platinum, palladium, nickel, and copper occupy an extensive space in the literature [45,46,47,48], while among the complexes, those of the osmium are the most promising [48,49,50].

On the other hand, aromatic nitro derivatives are compounds that are relatively easily obtained by aromatic electrophilic substitution, being then converted to substances with higher use value [36,37]. The reduction of aromatic nitro derivatives by various chemical, electrochemical or catalytic techniques is followed by inherent separation and purification processes [38,39,40].

The utilization of catalytic membranes solves the problem of impurity of the final product as well as avoiding the additional stages of separation, concentration, and purification [41,42].

In a particular case, the reduction of 5–nitrobenzimidazole raises supplementary issues due to the complexation capacity of metal ions coming from the chemical reduction of metals in acidic or basic medium, of its reduced stability in strong basic medium, as well as the low technical-economic efficiency of the separation of the reaction products.

Although 5–nitrobenzimidazole has applications as photographic material, an antioxidant in the rubber industry or in the construction of cadmium or bismuth copper sensors, its value increases greatly especially for the field of drugs, if it is transformed by reduction into 5–aminobenzimidazol.

The present paper presents the obtaining of composite polymeric membranes containing osmium obtained in situ, by reducing osmium tetroxide on cellulose acetate (CA), polysulfone (PSf) and polypropylene hollow fiber membranes (PP), for the reduction with molecular hydrogen of 5–nitrobenzimidazole to 5–aminobenzimidazole.

## 2. Materials and Methods

### 2.1. Reagents and Materials

The materials used in the presented work were of analytical grade. They were purchased from Merck (Merck KGaA, Darmstadt, Germany): sodium hydroxide, hydrochloric acid, and from Sigma-Aldrich (Merck KGaA, Darmstadt, Germany): 5–nitrobenzimidayole, t-Bu-OH and osmium tetroxide.

The solvents were: N,N’dimethylformamide (DMF), N methylpyrolidone (NMP) (ACS puriss reagent, Sigma-Aldrich, Merck, Redox Lab Supplies Com SRL, Bucharest, Romania), and ethyl alcohol (E) (Supelco^®^, Merck, Redox Lab Supplies Com SRL, Bucharest, Romania).

The purified water characterized by 18.2 μS/cm conductivity was obtained with a RO Millipore system (MilliQ® Direct 8 RO Water Purification System, Merck, Darmstadt, Germany).

**Figure 3:**
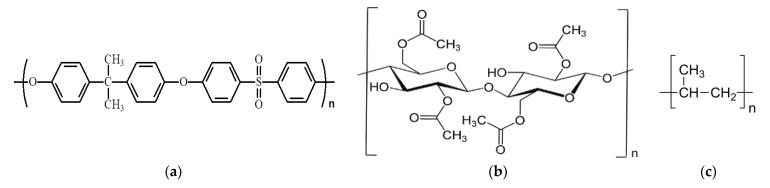
The chemical structure of (**a**) polysulfone (PSf), (**b**) cellulose acetate, and (**c**) polypropylene (PP).

The polysulfone (PSf) (Figure 3a) used here was a high-viscosity BASF type (S6010—BASF, BTC Europe GmbH, Budapest, Hungary).

Cellulose acetate (CA) (Figure 3b) powder, respectively, Mn = 50,000 g/mol, ρ = 1.3 g/cm^3^ (Sigma-Aldrich, Merck KGaA, Darmstadt, Germany).

The hollow polypropylene (Figure 3c) fibers used as support for the membranes were provided by GOST Ltd. (Perugia, Italy) [51,52].

Asymmetric cellulose acetate membranes were obtained by phase-inversion method, using immersion-precipitation techniques. The filming of the cellulose acetate solution in N-methylpyrolidine (NMP) was performed at room temperature and humidity, using a certain amount of 10% polymer solution by applying it directly to a smooth glass surface (intended for thin-layer chromatography) to a thickness of 500 μm. The immersion in the ethanol–water (EW) coagulation bath in a 1:2 volume ratio was performed after a predetermined time [53,54].

Asymmetric polysulfone membranes were also obtained by phase-inversion method, using immersion-precipitation techniques. The 10% polysulfone casting solution in dimethylformamide (DMF) was performed at room temperature (24 ± 1 °C) and humidity (61 ± 1%), using a certain amount of solution and applying it directly to a smooth glass surface (intended for thin-layer chromatography) to a thickness of 500μm. Immersion in the ethanol–water (EW) coagulation bath, in a 1:1 volume ratio, was performed after a predetermined time [55,56].

### 2.2. Methods

#### 2.2.1. Preparation of the Osmium Nanoparticles Composite Polymer Membrane

**Figure 4:**
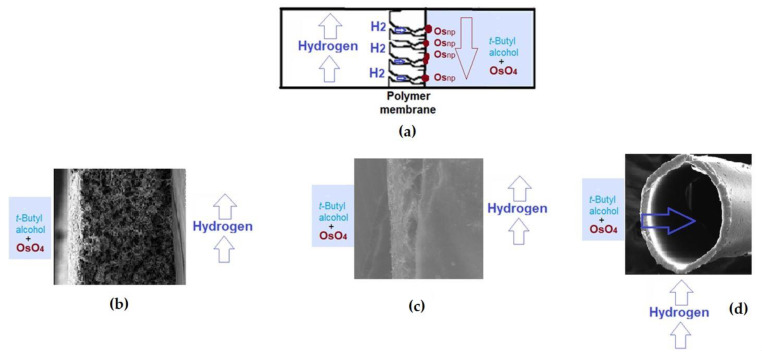
The schematic presentation for obtaining the composite osmium nanoparticles (Os_np_)–polymer membranes: (**a**) general scheme; and cases: (**b**) cellulose acetate membrane; (**c**) polysulfone membrane; (**d**) polypropylene hollow fiber membrane.

**Figure 5:**
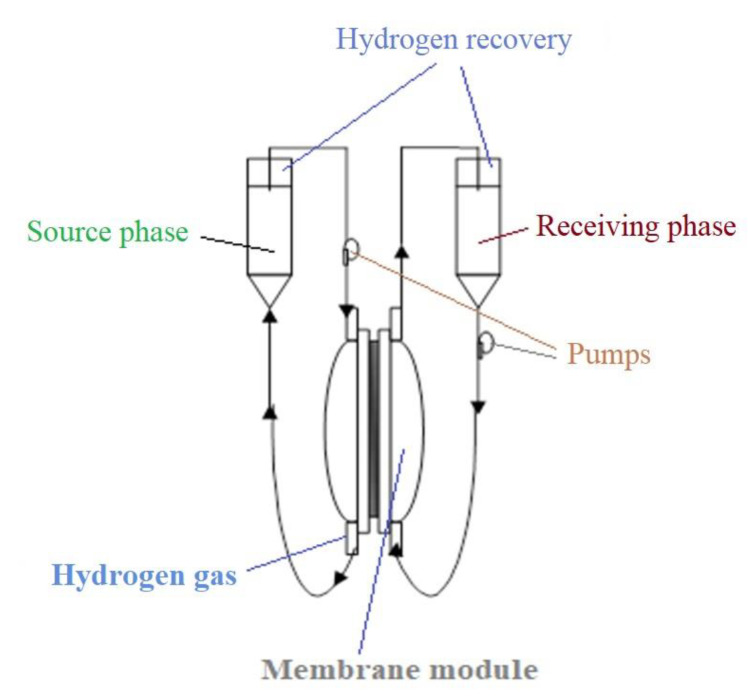
The schematic presentation of the reduction installation of 5–nitrobenzimidazole to 5–aminobenzimidazole.

The solution of osmium tetroxide is obtained by breaking an ampoule containing 1 g OsO_4_ in 250 mL tert–butyl alcohol, at room temperature, when a transparent light-yellow solution is obtained. The solution is placed, as the source phase, in a permeation cell (Figure 4 and Figure 5) separated by the considered membrane: polysulfone, cellulose acetate or polypropylene (Figure 4b–d). In the second compartment of the permeation cell, molecular hydrogen is circulated under reduced pressure. The polymeric membrane is constituted as a contactor in which the reduction of osmium tetroxide to metallic osmium takes place:

OsO_4_ + 4 H_2_ → Os + 4 H_2_O (1)

A composite membrane containing osmium nanoparticles is obtained—the polymeric membrane, on which the osmium layer is visible as an adhesive black deposit.

The nanocomposite osmium membranes samples were characterized by scanning electron microscopy (SEM), high-resolution SEM (HR–SEM), energy-dispersive spectroscopy analysis (EDAX), thermogravimetric analysis (TGA) and differential scanning calorimetry (DSC).

#### 2.2.2. Reduction of 5–Nitrobenzimidazole with Molecular Hydrogen in Membrane Reactor

The reduction of 5–nitrobenzimidazole is performed in a permeation cell (Figure 5) in which the source phase is a 0.5% 5–nitrobenzimidazole aqueous solution and the receiving phase is an aqueous solution of HCl 0.1 mol/L. Molecular hydrogen is introduced either by a hydrogen cylinder via specific pressure reducers, or by a gas pump, which takes over the hydrogen obtained by a reaction of a metal with an acid or base (Figure 2 and Figure 5).

Ar − NO_2_ + 3H_2_ → Ar − NH_2_ + 2H_2_O (2)

The nitro-derivative solution is constituted as the source phase and circulates at a constant flow of 100 mL/min on the membranes side, on which the osmium nanoparticles were deposited (Figure 4b–d—the back of the cellulose acetate and polysulfone membranes and the exterior of polypropylene hollow fiber membrane). The pH = 1 receptor solution circulates at a flow rate of 10 mL/min on the more compact surface (top surface) of the cellulose acetate and polysulfone membranes and inside the polypropylene hollow fiber membranes.

Molecular hydrogen is bubbled through a microporous frit at the base of the source phase with a variable flow rate provided by a high-performance reducer system (specific to chromatographic devices). Residual hydrogen from the system (found in both compartments of the membrane module) is recovered in impermeable and elastic polymeric containers. The experiments were repeated five times and the performed analyses allowed for an assessment of the deviation of the results, which was below 0.1%.

The fluxes from the source phase [57] were determined against the measured permeate mass within a determined time range, applying the following equation:

(3) $$J=\frac{M}{S\cdot t\;}\;(\mathrm{mg}⁄(m^{2}\;s))\;\mathrm{or}\;((\mathrm{mol})⁄(m^{2}\;s))$$

where: *M* is the permeate mass (g or mol), *S* is the effective surface of the membrane (m^2^), and *t* is the time (s) necessary to collect the permeate volume.

The extraction efficiency (*EE*%) for the species of interest using the concentration of the solutions [58] was calculated as follows:

(4) $$EE\left(\%\right)=\frac{\left(c_{0}-c_{f}\right)}{c_{0}}\cdot 100\;$$

where: *c_f_* is the final concentration of the solute (considered chemical species) and *c_0_* is the initial concentration of solute (considered chemical species).

The same extraction efficiency can also be computed based upon the absorbance of the solutions [59,60,61], as in:

(5) $$EE\left(\%\right)=\frac{\left(A_{0}-A_{s}\right)}{A_{0}}\;\cdot 100$$

where: *A_0_* is the initial absorbance of sample solution and *A_s_* is the current absorbance of the sample.

The conversion efficiency of the 5–nitrobenzimidazole reduction to 5–aminobenzimidazole is computed using expression (6), having a specific notation (*η*):

(6) $$\eta \left(\%\right)=\frac{\left(Ao{,}_{NAr}-Ai{,}_{NAr}\right)}{Ao_{NAr}}\;\cdot 100$$

where: *A_0,NAr_* is initial absorbance of 5–nitrobenzimidazole sample solution and *A_i,NAr_* is the current absorbance of 5–nitrobenzimidazole sample solution.

### 2.3. Equipment

The microscopy studies, SEM and HFSEM, were performed on a Hitachi S4500 system (Hitachi High-Technologies Europe GmbH, Krefeld, Germany). Thermal characterizations were performed on a Netzsch Thermal Analyzer (Netzsch—Gerätebau GmbH, Selb, Germany). The thermal analysis was run in a nitrogen atmosphere at 10 °C/min heating rate, from the room temperature (RT = 25 °C) up to 900 °C.

The UV–Vis analyses of the aqueous 5–nitrobenzimidazole solutions were performed on a Spectrometer CamSpec M550 (Spectronic CamSpec Ltd., Leeds, UK).

The UV–Vis spectra of the samples were recorded for a wavelength from 200 to 800 nm, at room temperature, using 10 mm quartz cells.

Spectroscopy Bruker Tensor 27 Fourier Transform Infra-Red (FTIR) with Diamond ATR (Bruker Scientific LLC, Billerica, MA, USA) was used to study the interactions between the chemicals used in the developed membranes. FTIR analysis was recorded in the range of 500 to 4000 cm^−1^.

The electrochemical analysis was followed up with a PARSTAT 2273 Potentiostat (Princeton Applied Research, AMETEK Inc., Oak Ridge, TN, USA). A setup with a glass cell with three electrodes has been used.

## 3. Results and Discussions

**Figure 6:**
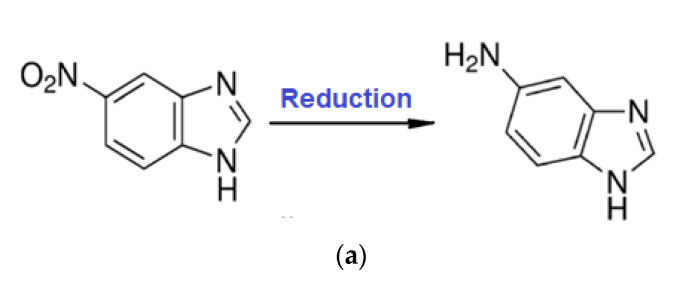

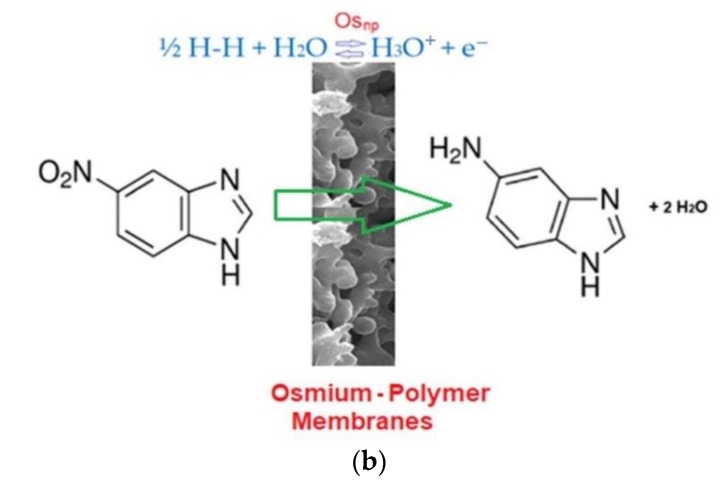
Reduction scheme of 5–nitrobenzimidazole to 5–aminobenzimidazole (**a**) and reduction scheme of 5–nitrobenzimidazole to 5–aminobenzimidazole on osmium–polymer membranes (Os–P) (**b**).

The study of 5–nitrobenzimidazole reduction is of particular importance, because the reaction product 5–aminobenzimidazole has much higher technical-economic value [59,61] (Figure 6a).

The novelty of the study consists in the reduction with hydrogen gas of the chosen aromatic derivative (Figure 6b) by using osmium nanoparticles-polymer composite membranes, obtained in situ by reduction (1) of osmium tetroxide with hydrogen gas on polymer membranes.

The study covers three objectives:

- Preparation of polymeric membranes containing osmium;
- The morphological and structural characterization of the prepared membranes;
- Reduction of 5–nitro benzimidazole to 5–aminobenzimidazole.

### 3.1. The Preparation and Characterization of Osmium–Polymer Membranes

The preparation of osmium–polymer membranes was made in a membrane contactor with three different types of membranes: cellulose acetate (CA), polysulfone (PSf), and hollow fiber polypropylene membrane (PP).

Their characteristics are presented in Table 1.

The cut-off determination (Molecular Weight Cut-Off or MWCO) was performed with a standard set of proteins using the formula in (5), for a retention of 95% [62,63]. Protein concentrations were determined by the Lowry method of protein dosing [64].

**Figure 7:**
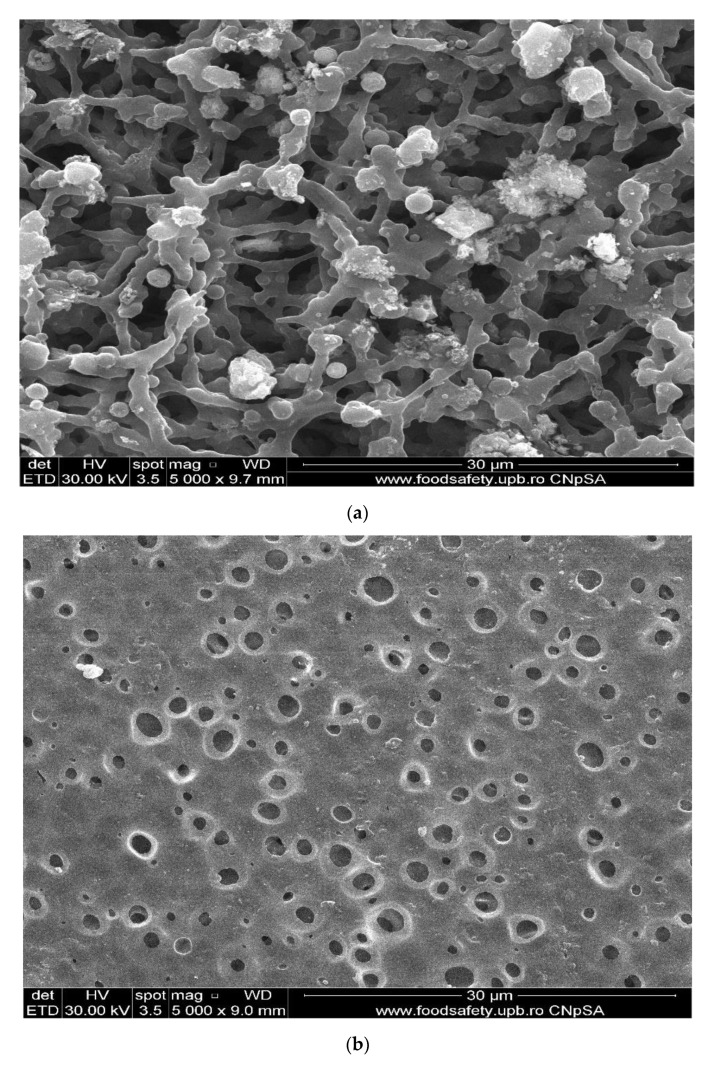

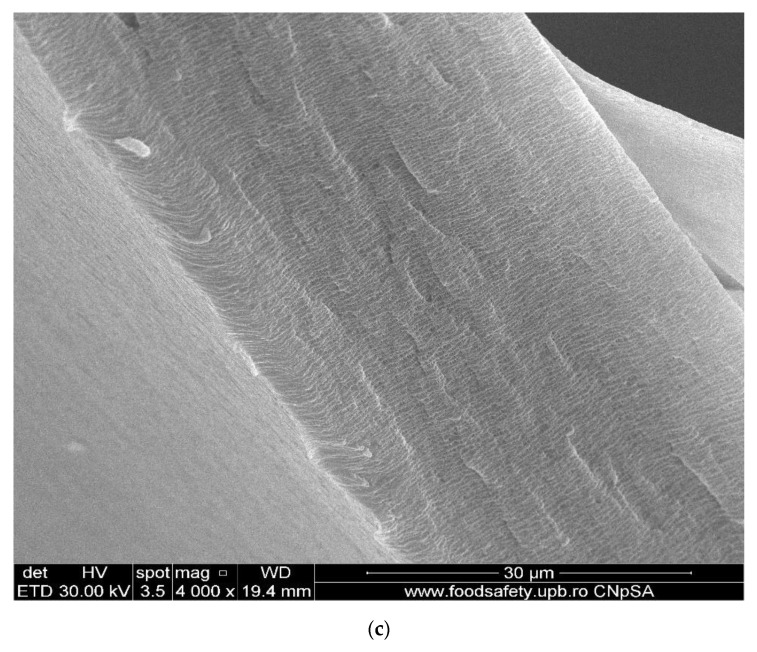
The bottom surface of the cellulose acetate (**a**) and the polysulfone (**b**) membranes, and cross section (**c**) of the polypropylene membranes.

The considered membranes constituted the contactor separation surface of the solution of 1 g Os/250 mL *tert-butyl alcohol*, for cellulose acetate membranes (CA), polysulfone (PSf) and 1 g/2.5 L hollow fiber polypropylene membranes. The flow of hydrogen through the installation, on the porous surface of the asymmetric cellulose acetate and polysulfone membrane (Figure 7a,b) and inside the symmetrical polypropylene membrane (Figure 7c), was 1 L/minute, at normal pressure.

After four hours of processing, the obtained osmium–polymer membranes are ready for the 5–nitrobenzimidazole reduction process.

### 3.2. Determination of Morpho-Structural Characteristics of Osmium–Polymer Membranes

Scanning electron microscopy (SEM), high-resolution SEM (HR–SEM), energy-dispersive spectroscopy (EDAX), and thermogravimetric analysis (TGA, DSC) were used to characterize osmium–polymer membranes.

#### 3.2.1. Scanning Electron Microscopy (SEM), High-Resolution SEM (HR–SEM) for the Osmium–Polymer Membranes

**Figure 8:**
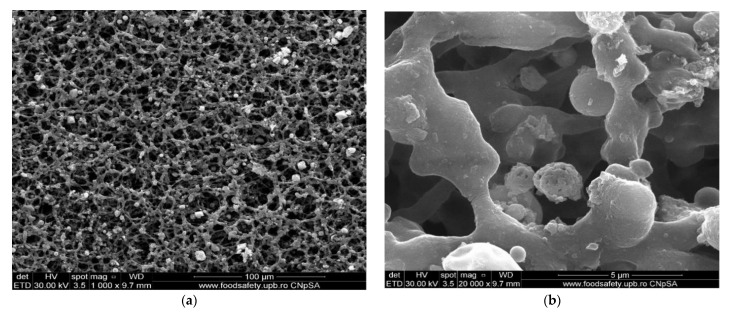

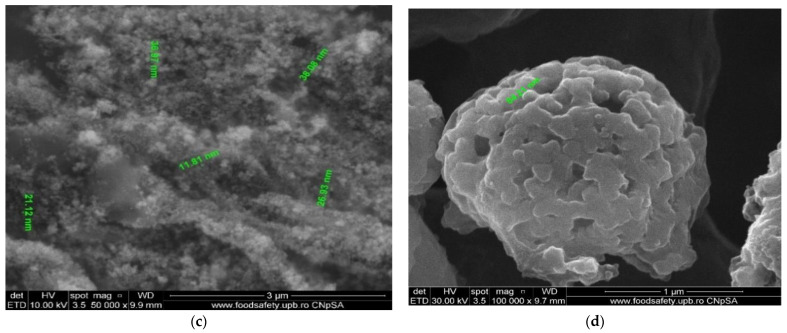
The scanning electron microscopy of the osmium–cellulose acetate membrane (Os–CA): membrane surface (**a**,**b**); cross section (**c**); and nanoparticle details (**d**).

**Figure 9:**
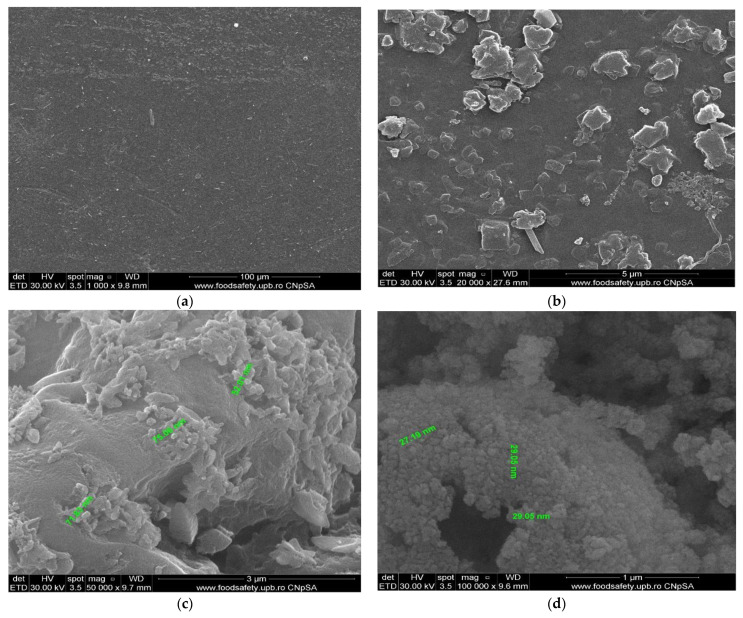
The scanning electron microscopy of the osmium–polysulfone membranes (Os–PSf): membrane surface (**a**,**b**); cross section and nanoparticle details (**c**,**d**).

**Figure 10:**
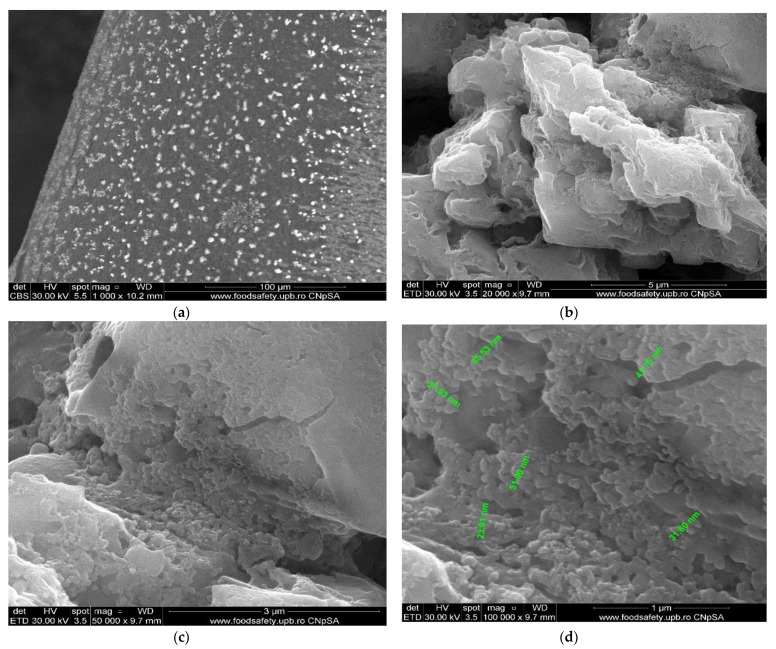
The scanning electron microscopy of the osmium–polypropylene membrane (Os–PP): membrane surface (**a**,**b**); cross section and nanoparticle details (**c**,**d**).

The membrane samples were washed with water and ethanol, dried in a vacuum oven, and then metallized with gold. The obtained images are presented as follows: osmium–cellulose acetate membrane (Os–CA) (Figure 8), osmium–polysulfone membranes (Os–PSf) (Figure 9), and the osmium–polypropylene membrane (Os–PP) (Figure 10) (see also Figure S1).

The first important observation, which refers to all three types of osmium–polymer membranes, is that osmium is obtained in the form of nanoparticles that aggregate (Figure 8, Figure 9 and Figure 10). The second general observation is that the osmium nanoparticles are obtained both on the surface and inside the membranes, as shown in details (b) and (c) of Figure 8, Figure 9 and Figure 10.

What is particular about each membrane can be seen in Figure 7, Figure 8 and Figure 9 and Figure 10a, as follows:

- The cellulose acetate membrane has a spongy structure (Figure 7a) in which the aggregates of osmium nanoparticles have spherical shapes that adhere to the walls of the micro-pores (Figure 8a);
- The polysulfone membrane (Figure 7b) has the micro-pores totally covered with osmium nanoparticles, aggregated in parallelepiped shapes (Figure 9a);
- The polypropylene membrane (Figure 7c) has in the micro-pores osmium nanoparticles aggregated in pyramids, which protrude from the surface of the polymeric support (Figure 10a).

The dimensions and shape of the osmium nanoparticles are observed in the details (d) of Figure 8, Figure 9 and Figure 10. In all cases, the shapes (at a magnitude of ×100,000) are spherical, their dimensions ranging between 15 and 55 nm.

#### 3.2.2. Thermal Analysis for the Osmium–Polymer Membranes

**Figure 11:**
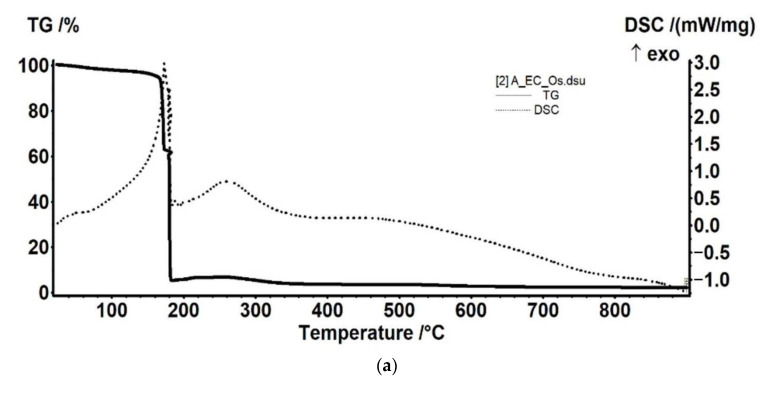

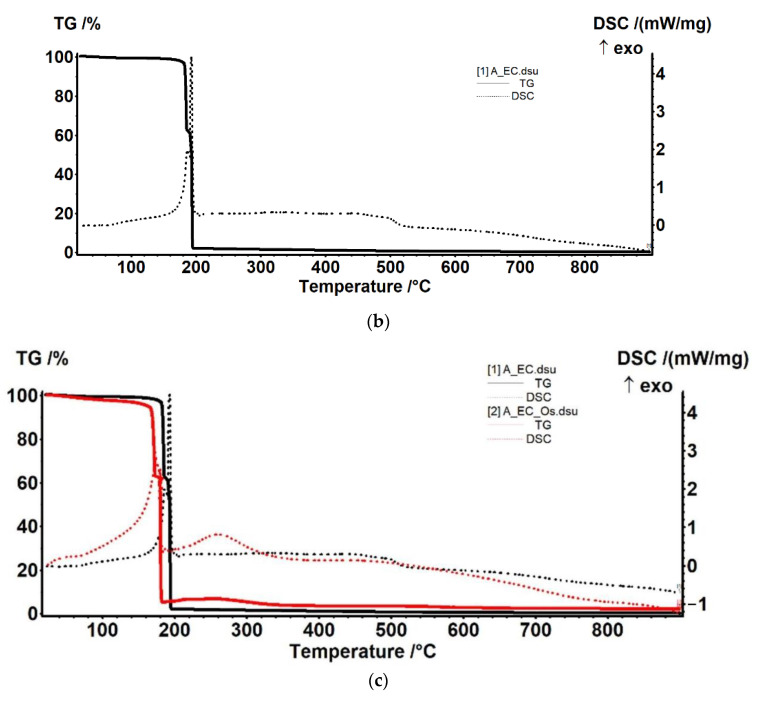
The thermal diagrams for: osmium–cellulose acetate membrane (Os–CA) (**a**); cellulose acetate (CA) (**b**); and their overlap (**c**).

**Figure 12:**
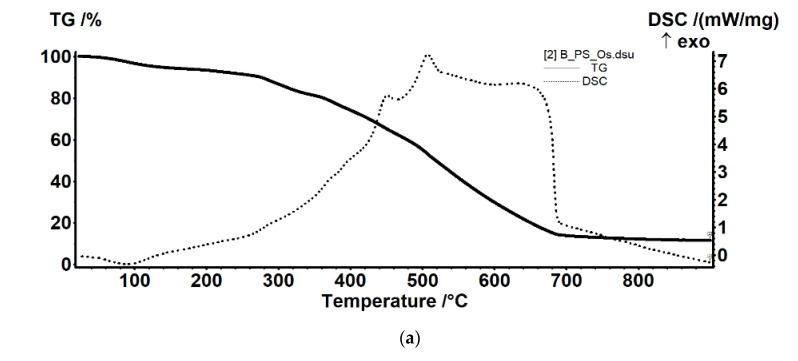

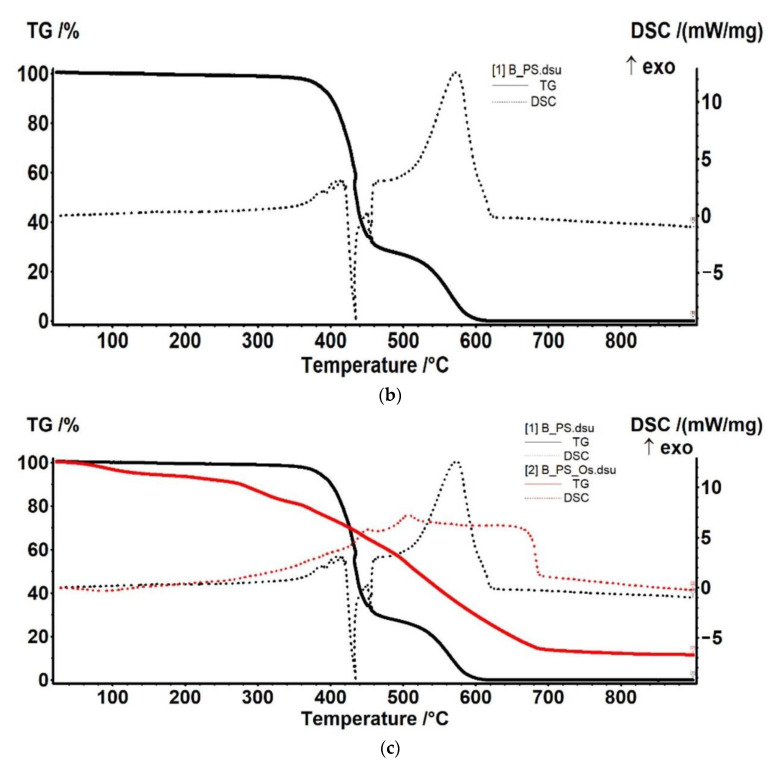
The thermal diagrams for: osmium–polysulfone membrane (Os–PSf) (**a**); polysulfone (PSF) (**b**); and their overlap (**c**).

**Figure 13:**
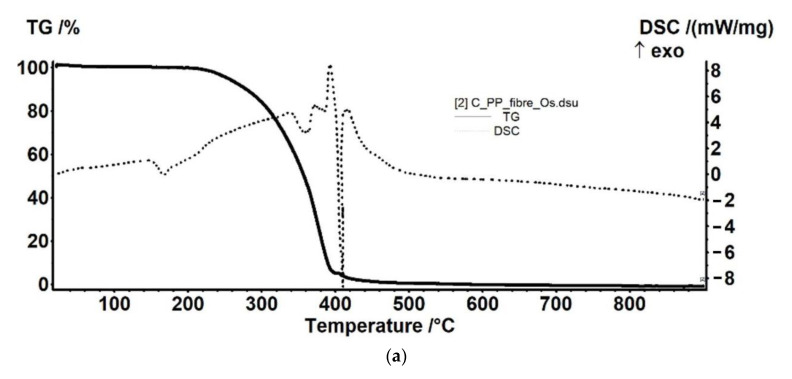

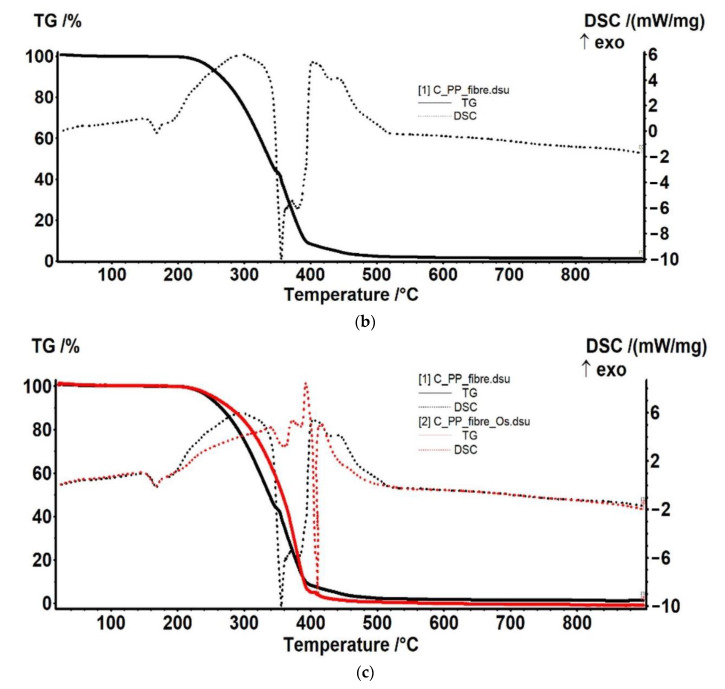
The thermal diagrams for: osmium–polypropylene membrane (Os–PP) (**a**); polypropylene hollow fiber membrane (PP) (**b**); and their overlap (**c**).

Thermal analysis of the osmium–polymeric (Os–P) membranes was performed by comparison with that of support polymeric membranes. The details of the thermal analysis that are presented in Figure 11, Figure 12 and Figure 13 took into account the possible thermal effects in the subsequent processing (see also Figures S2–S4).

The Os–CA membrane sample (Figure 11) has a higher percentage of volatile components evolved in early heating stage (up to 160 °C)—probably water or another solvent used in fabrication of the membrane. In addition, the thermal stability is lower, but only by ~10 °C. Most probably, after the organic part is burned away, Os will be oxidized to a volatile compound like OsO_4_, which is removed by further heating. Nevertheless, some additional residual mass is obtained, for Os–CA membrane sample (Figure 11a).

The sample loses 1.53% of its initial mass, up to 170 °C, the process being accompanied by a weak endothermic effect, with a minimum at 41.9 °C. This indicates that the sample is losing some residual water/solvent molecules from its structure (Figure 11a).

Between 170 and 200 °C, the CA membrane sample (Figure 11b) is oxidized quickly, the burning being a two-step process. In the first step, between 170 and 188 °C, the sample loses 36.26% of initial mass and the exothermic effect presents a peak at 185.7 °C. The second step, between 188 and 200 °C, is accompanied by a stronger exothermic effect, with maximum at 193.4 °C, the recorded mass loss being 60.26%.

After 200 °C, some residual carbonaceous mass is burned away with a mass change of 1.70%.

Between RT-160 °C, the sample loses 4.49% of initial mass. Most probably, the sample eliminates some residual water/solvent molecules from its structure, accompanied by an endothermic effect with minimum at 67 °C.

Between 160 and 190 °C, the Os–CA sample is oxidized quickly, the burning taking place in two steps. In the first step, 160–177 °C, the sample loses 33.08% of initial mass and the corresponding exothermic effect presents a peak at 172.9 °C. The second step, between 177 and 190 °C, is accompanied by a second exothermic effect, with maximum at 182.7 °C, the recorded mass loss being 57.08%.

After 190 °C, up to 260 °C, the sample gains 1.26% of its mass, the process being accompanied by an exothermic effect with maximum at 260.7 °C. This can be attributed to the oxidation of osmium.

The Os–PSf membrane sample (Figure 12a) is less stable than the PSf membrane (Figure 12b). The Os compound, impregnated on membrane, is decreasing the stability of the sample, and probably catalyzes various oxidative reactions. As the membrane undergoes such partial oxidation reactions, the TGA curve is quite different from simple PSf membrane sample. We detected no endothermic effect for the Os–PSf sample, and the total degradation is delayed, the residual mass also being higher.

The sample is thermally stable up to 340 °C, the recorded mass loss of 1.62% being due to some volatile residuals trapped into the membrane structure or oxidation of some impurities on the membrane surface. After 340 °C, the sample suffers a degradative–oxidative complex process. The mass loss in the temperature interval 340–450 °C is 63.68%. The process is accompanied by a series of overlapped exothermic effects, with peaks at 400.9 °C and 408.3 °C, which indicates oxidative reactions, followed by an endothermic effect with minimum at 435.1 °C, which indicates a decomposition.

Between 450 °C and 620 °C, the sample is losing 34.83% of initial mass, in an oxidative process, the corresponding exothermic effect, strong and asymmetric, presenting a peak at 574.3 °C.

The sample is losing 6.11% of initial mass in the interval RT-180 °C. The process is accompanied by an endothermic effect, with minimum at 89.3 °C, indicating that some water or solvent molecules trapped into the membrane structure are lost.

The derivative thermogravimetric (DTG) curve not shown here indicates that two distinct mass losses are occurring, as follows: 2.19% of mass loss, between 180 °C and 245 °C, and 10.40% mass loss from 245 °C to 350 °C. Both are accompanied by weak exothermic effects, shown on DSC curve like shoulders, e.g., at 288 °C. This indicates that the processes are predominantly oxidative, either Os or Os compounds accelerating the degradation of the membrane.

After 350 °C, the sample suffers a degradative–oxidative complex process, like the PSf sample, but on different reaction pathways, as the PSf is already degraded by previous processes. The mass loss in the temperature interval 340–470 °C is 19.66%. The process is accompanied by a series of overlapped exothermic effects, with main peak at 451.5 °C. A strong oxidative process takes place between 470 and 700 °C, when a mass loss of 47.87% is recorded. The process is accompanied by a strong, broad, asymmetric exothermic effect with maxima at 507.4 °C and at 629.5 °C.

The residual mass is 11.42% at 900 °C (black bit, nonmagnetic, friable).

The Os–PP membrane sample (Figure 13) is marginally more resistant to the heating. The presence of Os compounds is shielding the PP fibers, delaying the decomposition/oxidation with about 15 °C.

The PP membrane fibers (Figure 13b) are thermally stable up to 210 °C. The sample loses 0.53% of its initial mass, most probably due to some plasticizers or other impurities. In the RT-210 °C interval, a small endothermic effect can be observed on the DSC curve, with onset at 156.1 °C and a peak at 168.1 °C, which corresponds to the melting point of polypropylene.

In the temperature interval 210–400 °C, the main degradation process occurs, the recorded mass loss being 91.16%. On the DSC curve, the process is accompanied by a broad exothermic effect, with maximum at 296.3 °C. This corresponds to the oxidation of some smaller polymer branches. At 355.9 °C and 380.3 °C, two endothermic effects were recorded, corresponding to the breaking of covalent bonds and volatilization of low-mass products. This indicates that the sample undergoes a series of oxidative–degradative reactions, up to 400 °C.

The carbonaceous residual mass is burned away after 400 °C, when a mass loss of 6.33% is recorded. On the DSC curve, a strong, broad, asymmetric exothermic effect is recorded, with the maxima at 404 °C and 440.6 °C.

The Os–PP membrane fibers (Figure 13a) are thermally stable up to 210 °C, like the PP sample, the recorded mass loss being 0.53%. In the RT-210 °C interval, on the DSC curve, can be observed a small endothermic effect, with onset at 153 °C and peak at 167.5 °C, which corresponds to the melting point of polypropylene. The melting temperature is a few degrees lower because Os acts as an impurity.

In the temperature interval 210–400 °C, the main degradation process occurs, the recorded mass loss being 94.37%. The process is accompanied on the DSC curve by a broad exothermic effect, with maxima at 338.6, 374.2, and 393.2 °C. This corresponds to the oxidation of organic compound. It can be noticed that, in this case, the oxidative degradative processes start at higher temperatures (~15 °C higher).

Right after 400 °C, a decomposition process is recorded, accompanied by an endothermic effect on the DSC curve, with minimum at 410.6 °C. The residual carbonaceous mass is burned away, the corresponding exothermic effect presenting a peak at 417.6 °C.

#### 3.2.3. Compositional Characterization of Membranes

Energy-dispersive spectroscopy analysis (EDAX) for the osmium–polymer membranes and Fourier Transform Infra-Red spectroscopy were performed in order to be able to correlate the process performances with the composition of the prepared osmium–polymer membranes (Os–P).

**Figure 14:**
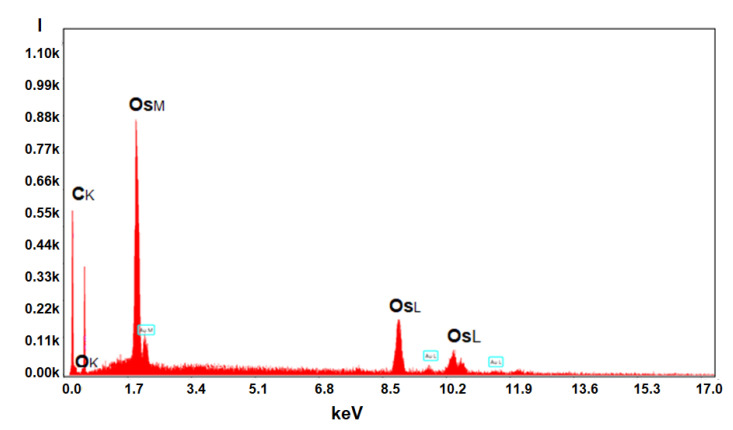
Formal energy-dispersive spectroscopy analysis (EDAX) for the osmium–polymer membranes.

**Figure 15:**
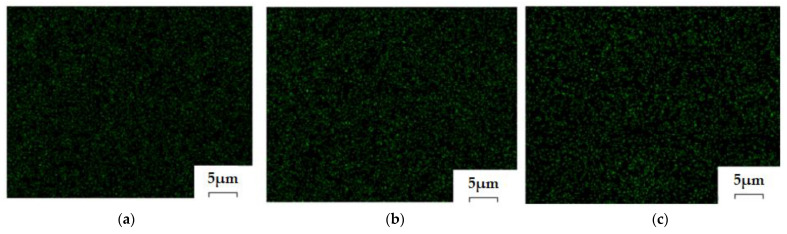
Osmium distribution images by energy-dispersive spectroscopy analysis (EDAX) for the osmium–polymer-obtained membranes: Os–CA membrane (**a**); Os–PSf membrane (**b**); OS–PP membrane (**c**).

**Figure 16:**
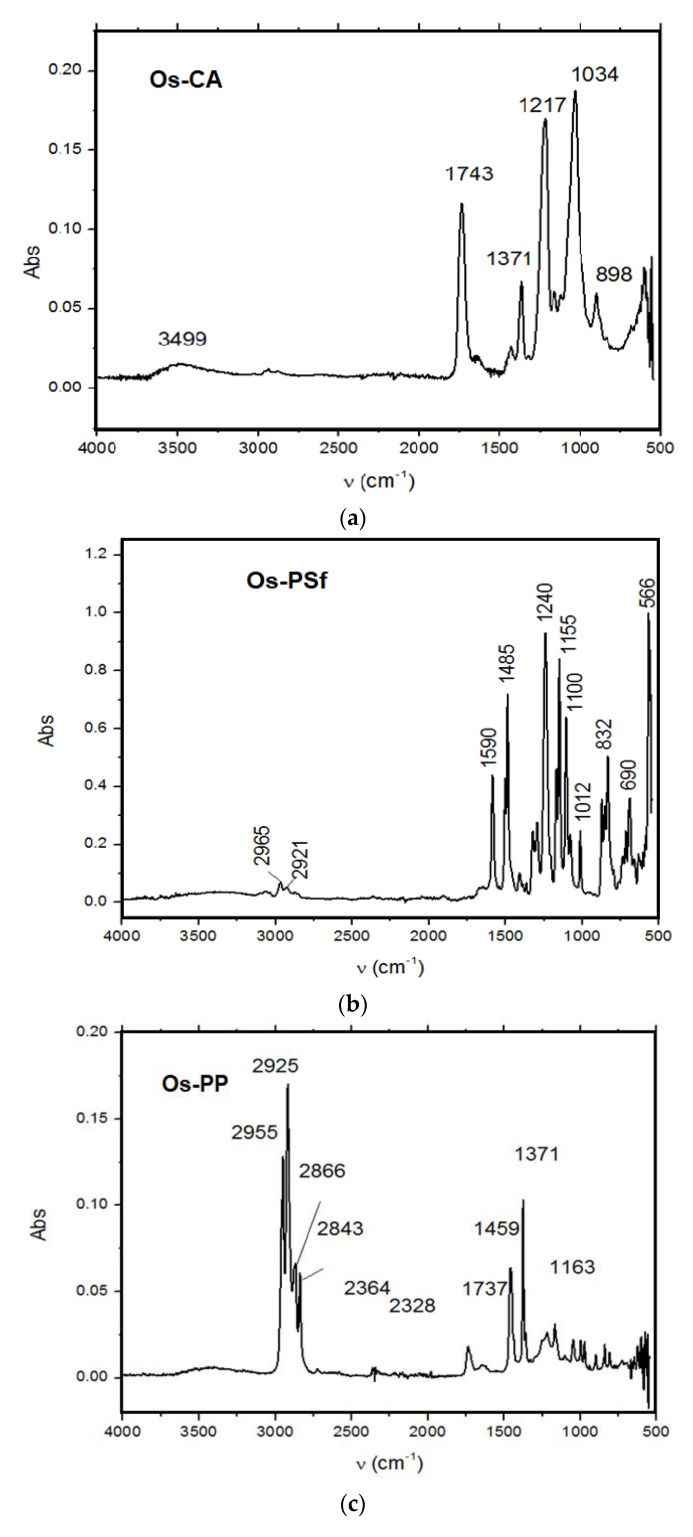
Fourier Transform Infra-Red spectroscopy (FTIR) spectrum of: (**a**) osmium–cellulose acetate membrane (OS–CA); (**b**) osmium–polysulfone membrane (Os–PSf); and (**c**) osmium–polypropylene hollow fiber membranes (Os–PP).

Energy-dispersive spectroscopy analysis (EDAX) for the osmium–polymer membranes provides the surface composition and osmium distribution; its appearance being shown in Figure 14, Figure 15 and Figure 16 (see also Figure S5).

Table 2 presents the results of the surface analysis for all three types of membrane prepared: osmium–cellulose acetate (Os–CA), osmium–polysulfone (Os–PSf), and osmium–hollow fiber polypropylene membrane (Os–PP).

The compositional analysis of the membrane surface shows the important differences between the three membrane materials, as follows:

- The carbon atoms in mass percentage differ greatly among the three membrane surfaces, but in number they are relatively closer;
- Oxygen atoms on the Os–PP membrane surface (coming from water or alcohol molecules or adsorbed in the membrane preparation process) are much less numerous than on the Os–PSf membrane, but especially on the Os–CA membrane;
- Osmium atoms are important in gravimetric weight on all three types of membranes, but from an atomic point of view they are below 10%;
- The sulfur atoms appear in a significant percentage only on the polysulfone membranes.

Energy-dispersive spectroscopy analysis (EDAX) is a local analysis, which depends a lot on the selected area on the surface of the membrane and its spatial orientation, and therefore, the results show important errors (Table 2). Nevertheless, the information provided by EDAX is qualitatively very important, the type of atoms on the surface being indicated with high fidelity.

For this reason, the data provided by EDAX must be correlated with a complementary method of the highest confidence, regarding the composition in terms of bonds and functions of polymeric materials, Fourier Transform Infra-Red (FTIR) spectroscopy (Figure 16).

The Fourier Transform Infra-Red (FTIR) spectra obtained for the three composite membranes do not show differences in the position of the absorption bands compared to those of the base polymers [52,53,54].

The obtained spectra reveal the functions and bonds specific to the polymer and important for each obtained osmium–polymer membrane, as follows:

- For Os–CA membrane the carbonyl function of the ester (*v* C = O 1743 cm^−1^);
- For Os–PSf membrane sulfone function (*v* S = O occurs in the range 1100–1330 cm^−1^);
- For OS–PP membrane carbon–hydrogen bond (*v* C–H occurs in the range 2840–2955 cm^−1^), but also the slight “shoulder” of the hydrogen bond given by the traces of adsorbed water (3300–3600 cm^−1^).

For the present study, the importance of the superficial interaction is great and for these three polymers with very different hydrophilicity, the following were chosen: polypropylene (hydrophobic), polysulfone (very little hydrophilic), and cellulose acetate (hydrophilic).

### 3.3. The Performances of Osmium–Polymer Membranes (Os-P) in the Process of Reducing 5–Nitrobenzimidazole to 5–Aminobenzimidazole

The reduction process of 5–nitrobenzimidazolului to 5–aminobenzimidazol according to reaction (Figure 6) was performed in an installation with a usable membrane area of 100 cm^2^, for the osmium–cellulose acetate membrane (Os–CA), or the osmium–polysulfone membrane (Os–PSf), and 10,000 cm^2^ for osmium polypropylene hollow fiber membranes (Os–PP). The processed solution volumes of 5–aminobenzimidazol (0.5 g/L) were: 1 L for the osmium–cellulose acetate membrane (Os–CA) or the osmium–polysulfone membrane (Os–PSf), and 10 L for osmium–polypropylene hollow fiber membranes (Os–PP). The volume of hydrogen circulated through the source phase (Figure 5) of the installation has a variable flow rate (Q) between 0.5 and 3.0 L/min hydrogen under normal temperature and pressure conditions.

**Figure 17:**
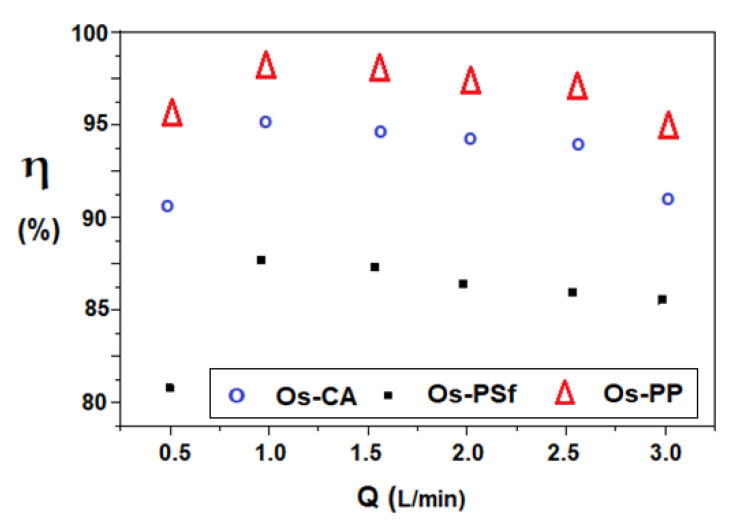
Variation of the conversion of 5–aminobenzimidazol according to the molecular hydrogen flow through the installation, for the prepared osmium–polymer (Os–P) membranes.

**Figure 18:**
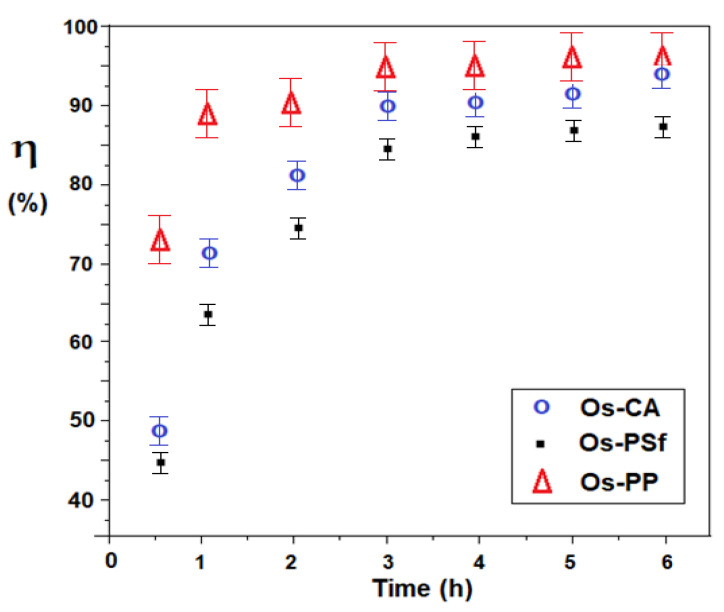
Variation of the conversion of 5–nitrobenzimidazol to 5–aminobenzimidazol depending on the operating time, for osmium–polymer (Os–P) prepared membranes.

**Figure 19:**
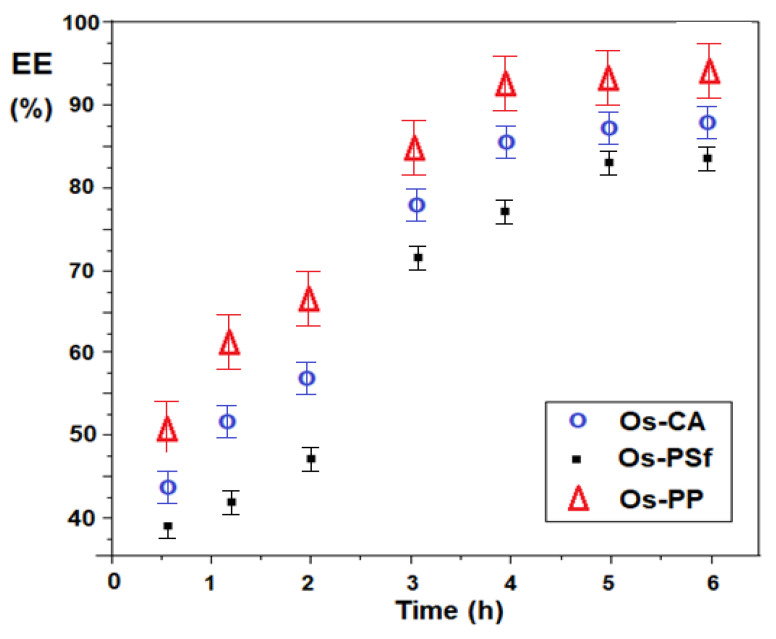
Efficiency of 5–aminobenzimidazol separation depending on the operating time, for the prepared osmium–polymer (Os–P) membranes.

**Figure 20:**
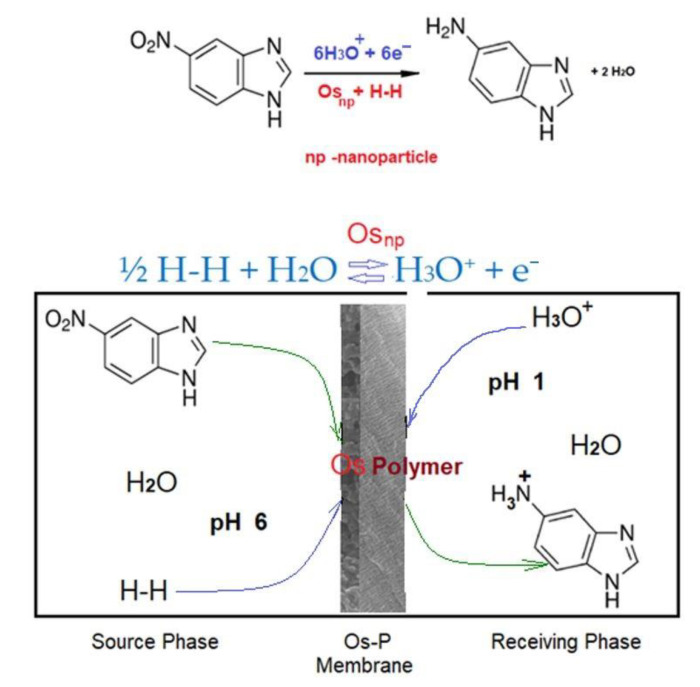
Proposed mechanism for transformation of 5–nitrobenzimidazol to 5–aminobenzimidazol in the reaction system using osmium–polymer (Os–P) membrane.

The 5–nitrobenzimidazol solution is the source phase (SP) and has a pH (pH = 6.0) determined by the presence of the chosen organic compound in pure water. The receiving phase is formed of HCl solution 0.1 mol/L (pH = 1). The receiving phase has a volume of 100 mL, for the osmium–cellulose acetate membrane (OS-CA) or the osmium–polysulfone membrane (Os–PSf), and 1.0 L for osmium–polypropylene hollow fiber membranes (Os–PP). The results of the study are presented in Figure 17, Figure 18, Figure 19 and Figure 20.

#### 3.3.1. The Influence of Hydrogen Flow in the Reduction Process of 5–Nitrobenzimidazol to 5–Aminobenzimidazol

Composite membranes have flow performances close to those of the support membranes (Table 3). The absence of an additional hydraulic resistance contributes to the operation in good conditions of the permeator. The fluxes of the utilized support membranes are specific to microfiltration, but approach those of ultrafiltration after the deposition of osmium nanoparticles. The operation of the permeator, in the studied cases, is done at atmospheric pressure.

Figure 17 shows the results of reducing 5–nitrobenzimidazol to 5–aminobenzimidazol for a variable hydrogen flow rate (0.5–3.0 L/minute), at an operating time of six hours, with the source phase solution of 5–aminobenzimidazol (0.5 g/L, pH = 6.0) and the receiving phase solution of HCl 0.1 mol/L (pH = 1).

The results show over 80% conversion for all three types of membrane, along the entire range of hydrogen flows studied. The optimum working flow is 1.0–1.5 L/min for all membranes, but although the conversion varies, it respects the order: η _Os-PP_ > η _Os-CA_ > η _Os-PSf_ throughout the entire interval.

#### 3.3.2. The Conversion of 5–Nitrobenzimidazol to 5–Aminobenzimidazol Depending on Time and Nature of the Osmium–Polymer (Os–P) Membrane

This conversion variation is presented in Figure 18.

In the first two operating hours, the conversion evolves rapidly, especially for the osmium–polypropylene (Os–PP) membrane, after which there is a significant reduction, leading to a plateau, especially to the osmium–polysulfone membrane (OS–PSf). Throughout the six hours of operation, the conversion follows the order: η _Os–PP_ > η _Os–CA_ > η _Os–PSf_.

The obtained result has two explanations: the osmium–polypropylene membrane has a much larger working surface (10,000 cm^2^), and in the case of the two membranes with equal surfaces (100 cm^2^), the one based on cellulose acetate better interacts with the aqueous medium, being more hydrophilic than the membrane based on polysulfone. After six operating hours, the conversion reaches a maximum, and therefore it is recommended to avoid exceeding this working time.

#### 3.3.3. The Evolution of the Efficiency of 5–Aminobenzimidazol Separation over Time and Depending on the Nature of the Osmium–Polymer (Os–P) Membrane

The efficiency of membrane separation of 5–aminobenzimidazole depending on the time and nature of the osmium–polymer (Os–P) membrane (Figure 19) follows the variation of the conversion of 5–nitrobenzimidazol to 5–aminobenzimidazol, being slightly lower in time, because the transfer of 5–aminobenzimidazol through membranes depends on its accumulation in the source phase in which the reduction takes place.

Throughout the six hours of operation, the efficiency of the separation respects the order: EE _Os–PP_ > EE _Os–CA_ > EE _Os–PSf_.

#### 3.3.4. Proposal of a Mechanism for the Transformation of 5–Nitrobenzimidazole to 5–Aminobenzimidazole in the Osmium–Polymer (Os–P) Membrane Reaction System

When elaborating the proposal mechanism of reaction and separation (Figure 20) of 5–nitrobenzimidazol by transformation into 5–aminobenzimidazol, in the reaction system with osmium–polymer membrane (Os–P), the following were taken into account:

- The conversion of 5–nitrobenzimidazole to 5–aminobenzimidazole in the reaction system with osmium–polymer membrane (Os–P) depends on the nature of the polymer;
- The conversion of 5–nitrobenzimidazol to 5–aminobenzimidazol in the reaction system with osmium–polymer membrane (Os–P) is slightly dependent on the hydrogen flow in the system;
- The efficiency of 5–aminobenzimidazol separation depends on the operating time, being correlated with the conversion of 5–nitrobenzimidazol to 5–aminobenzimidazol, in the reaction system with osmium–polymer membrane (Os–P);
- The working pH is imposed to 6 in the source phase (SP) and to 1 in the receiving phase (RP).

Figure 20 presents the main aspects of the mechanism of transformation of 5–nitrobenzimidazole to 5–aminobenzimidazole in the reaction system with osmium–polymer membrane (Os–P).

The most important aspect of the reduction of 5–nitrobenzimidazol to 5–aminobenzimidazol in the osmium–polymer (Os–P) membrane reaction system is the complex interaction of the organic compound with the molecular or atomic hydrogen containing osmium nanoparticles, on the polymeric membrane.

Of course, the hydrogenation of nitro derivatives or related compounds with hydrogen gas in a non-aqueous heterogeneous medium is widely studied and accepted, especially when osmium complexes with various ligands are used as catalysts [65,66]. Also, the reduction of nitro derivatives in aqueous medium with atomic hydrogen (proton and electron) coming from a metal in contact with an acid or base is very much studied [67].

The mechanism illustrated in Figure 20 proposes the reduction of 5–nitrobenzimidazol to 5–aminobenzimidazol on a membrane, in an aqueous medium, by the contact between the complex of the organic compound containing osmium nanoparticles deposited on a polymer and the molecular hydrogen in equilibrium with hydronium ions (the protons in the aqueous solution).

Of course, the most important problem of the mechanism is the fact that, theoretically, 5–nitrobenzimidazol can be reduced with molecular hydrogen activated by osmium nanoparticles or with the proton in the solution, which can take up an electron on the osmium nanoparticles in the aqueous osmium–polymer membrane system.

This problem of the reaction mechanism is proposed to be solved by a study of reduction of 5–nitrobenzimidazol to 5–aminobenzimidazol on an osmium–polymer membrane by using gaseous deuterium. By determining whether 5–aminobenzimidazol contains deuterium, the proposed mechanism can be finalized.

## 4. Conclusions

The paper presents the results obtained at the reduction of 5–nitrobenzimidazole by transformation into 5–aminobenzimidazole, in the reaction system with osmium–polymer membrane (Os–P) with molecular hydrogen, in an aqueous membrane system, with pH = 6 in the source phase and pH = 1 for the receiving phase.

This study opens the research direction of metallic osmium nanoparticles–polymer membranes to redox processes (reduction or oxidation) of organic compounds of biological interest that should not be contaminated with metal ions.

The osmium–polymer membranes (OS–P) were obtained using cellulose acetate membranes and polysulfone (PSf) membranes as support, obtained by phase inversion and commercial polypropylene hollow fiber (PP). The osmium in the form of nanoparticles was generated by the reduction reaction of osmium tetroxide in tert–butyl alcohol with molecular hydrogen.

The membranes obtained, based on osmium–cellulose acetate (OS–CA), osmium–polysulfone (Os–PSf), and osmium–polypropylene hollow fiber (Os–PP) membranes, were characterized from a morphological and structural point of view, using scanning electron microscopy (SEM), high-resolution SEM (HR–SEM), energy-dispersive spectroscopy analysis (EDAX), and thermogravimetric analysis (TGA, DSC).

The process performance was tested at reduction of 5–nitrobenzimidazol solution 0.5 g/L to 5–aminobenzimidazol with molecular hydrogen, by varying the nature and surface of the membrane, the molecular hydrogen flow, and the operating time.

The results obtained show that:

- The conversion of 5–nitrobenzimidazol to 5–aminobenzimidazol in the reaction system with osmium–polymer (Os–P) membrane depends on the nature of the polymer;
- The conversion of 5–nitrobenzimidazol to 5–aminobenzimidazol in the reaction system with osmium–polymer (Os–P) membrane is slightly independent of the hydrogen flow in the system;
- The efficiency of 5–aminobenzimidazol separation depends on the operating time, being correlated with the conversion of 5–nitrobenzimidazol to 5–aminobenzimidazol, in the reaction system with osmium–polymer membrane (Os–P).

Both the 5–aminobenzimidazol separation efficiency (EE) and the 5–nitrobenzimidazol to 5–aminobenzimidazol conversion efficiency (η) vary in the same order: EE _Os–PSf_ ≤ EE _Os–CA_ ≤ EE _Os–PP_ and, respectively, η _Os–PSf_ ≤ η _Os–CA_ ≤ η _Os–PP_.

Aspects of the possible mechanism of conversion of 5–nitrobenzimidazole to 5–aminobenzimidazole with hydrogen gas in the reaction system with osmium–polymer membrane (Os–P) are presented and a proposal is made to solve it by using deuterium (^2^H or D) instead of hydrogen or heavy water (D_2_O) as the reaction medium.

## Figures and Tables

**Table 1:** The characteristics of the membrane supports.

Membrane	Structure	Surface Area (cm^2^)	Porosity (%)	Pore Dimension or Cut-Off (MWCO)
Cellulose acetate (CA)	asymmetrical	100	78 + 3	68,000 (BSA) *
Polysulfone (PSf)	asymmetrical	100	65 + 4	34,500 (P) **
Hollow fiber poly-propylene membrane (PP)	symmetrical	10,000	40 + 2	0.002–0.2 µm

(*) Bovine Serum Albumin (BSA); (**) Pepsin (P).

**Table 2:** Energy-dispersive spectroscopy analysis (EDAX) for the osmium–polymer membranes.

Osmium–Polymer Membranes	Cellulose Acetate (Os–CA)			Polysulfone (Os–PSf)			Polypropylene (Os–PP)		
Surface Composition	Weight (%)	Atomic (%)	Error (%)	Weight (%)	Atomic (%)	Error (%)	Weight (%)	Atomic (%)	Error (%)
C K	34.47	58.44	12.24	29.57	66.81	14.9	49.36	90.32	9.44
O K	29.67	37.73	15.49	8.03	13.60	22.93	2.89	4.05	29.4
Os L	35.86	3.83	8.59	47.17	6.68	6.35	47.75	5.63	11.12
S K	–	–	–	15.23	12.91	8.11	–	–	–

**Table 3:** The flow characteristics of the composite and support membranes.

Pressure (atm)	Polymer Membrane Flux (J) (L/m^2^h)					
	Cellulose Acetate (CA)		Polysulfone (PSf)		Hollow Fiber Polypropylene Membrane (PP)	
	Support	Composite	Support	Composite	Support	Composite
1.5	23.3	21.5	18.9	17.7	11.4	9.7
2.0	31.7	28.4	23.7	20.1	13.8	10.9
2.5	38.9	35.5	27.8	24.5	15.1	13.1

## Data Availability

Not applicable.

## Supplementary Materials

The following are available online at https://www.mdpi.com/article/10.3390/membranes11080633/s1, Figure S1: The scanning electron microscopy on the cross-section of the studied membranes, Figure S2: The thermal diagrams for osmium-cellulose acetate membrane (Os–CA) and cellulose acetate (CA), Figure S3: The thermal diagrams for osmium-polysulfone membrane (Os–PSf) and polysulfone (PSF), Figure S4: The thermal diagrams for osmium-polypropylene membrane (Os–PP) and hollow fiber polypropylene membrane (PP), Figure S5. Formal energy dispersive spectroscopy analysis (EDAX) for the surface distribution of osmium on the studied membranes.

## Abbreviations

(CA),	Cellulose acetate
(PSf),	Polysulfone
(PPM),	Polypropylene hollow fiber membranes
(t–Bu–OH),	tert–Butyl alcohol
(Os–P),	Composite osmium–polymer membrane
(Os–CA),	Osmium–cellulose acetate membrane
(Os–PSf),	Osmium–polysulfone membranes
(Os–PP),	Osmium–polypropylene membrane
(SEM),	Scanning electron microscopy
(HR–SEM),	High-resolution SEM
(EDAX),	Energy-dispersive spectroscopy analysis
(FTIR),	Fourier Transform Infra-Red spectroscopy
(TGA),	Thermogravimetric analysis
(DSC),	Differential scanning calorimetry
(EE),	Separation efficiency
(η)	Conversion efficiency

## References

[1] Stannett V.T., Koros W.J., Paul D.R., Lonsdale H.K., Baker R.W. (1979). Recent advances in membrane science and technology. Chemistry.

[2] Kammermeyer K. (1976). Technical Gas Permeation Processes. Chem. Ing. Tech..

[3] Kesting R. (1988). Synthetic polymeric membranes. J. Colloid Interface Sci..

[4] Mulder M. (1996). Basic Principles of Membrane Technology.

[5] Baker W. (2012). Membrane Technology and Applications.

[6] Guizard C., Rios G. (1996). Chapter 12 Transport and fouling phenomena in liquid phase separation with inorganic and hybrid membranes. Membr. Sci. Technol..

[7] Merkel T.C., Freeman B.D., Spontak R.J., He Z., Pinnau I., Meakin P., Hill A.J. (2002). Ultrapermeable, Reverse-selective nanocomposite membranes. Science.

[8] Bazhenov S.D., Bildyukevich A.V., Volkov A.V. (2018). Gas-liquid hollow fiber membrane contactors for different applications. Fibers.

[9] Iulianelli A., Drioli E. (2020). Membrane engineering: Latest advancements in gas separation and pre-treatment processes, petrochemical industry and refinery, and future perspectives in emerging applications. Fuel Process. Technol..

[10] Mulder M., Crespo J.G., Böddeker K.W. (1994). The Use of Membrane Processes in Environmental Problems. An Introduction. Membrane Processes in Separation and Purification.

[11] Drioli E., Stankiewicz A.I., Macedonio F. (2011). Membrane engineering in process intensification—An overview. J. Membr. Sci..

[12] Bernardoa P., Drioli E. (2010). Membrane Gas Separation Progresses for Process Intensification Strategy in the Petrochemical Industry. Pet. Chem..

[13] Hradil J., Krystl V., Hrabanek P., Bernauer B., Kocirık M. (2004). Heterogeneous membranes based on polymeric adsorbents for separation of small molecules. React. Funct. Polym..

[14] Stepniak P., Lainer B., Chmurski K., Jurczak J. (2017). pH-Controlled recognition of amino acids by urea derivatives of β-cyclodextrin. RSC Adv..

[15] Kyne G.M., Light M.E., Hursthouse M.B., De Mendoza J., Kilburn J.D. (2001). Enantioselective amino acid recognition using acyclicthiourea receptors. J. Chem. Soc. Perkin Trans..

[16] Strathmann H., Giorno L., Drioli E. (2011). Introduction to Membrane Science and Technology.

[17] Jones M.N. (1999). Surfactants in membrane solubilization. Int. J. Pharm..

[18] Garavand F., Madadlou A. (2014). Recovery of phenolic compounds from effluents by a microemulsion liquid membrane (MLM) extractor. Colloids Surf. A.

[19] Atlaskin A.A., Kryuchkov S.S., Yanbikov N.R., Smorodin K.A., Petukhov A.N., Trubyanov M.M., Vorotyntsev V.M., Vorotyntsev I.V. (2020). Comprehensive experimental study of acid gases removal process by membrane-assisted gas absorption using imidazolium ionic liquids solutions absorbent. Sep. Pur. Technol..

[20] Tishchenko G., Luetzow K., Schauer J., Albrecht W., Bleha M. (2001). Purification of polymer nanoparticles by diafiltration with polysulfone/hydrophilic polymer blend membranes. Sep. Pur. Technol..

[21] Xiong L., Manthiram A. (2005). Nanostructured Pt–M/C (M = Fe and Co) catalysts prepared by a microemulsion method for oxygen reduction in proton exchange membrane fuel cells. Electrochim. Acta.

[22] Balta S., Sotto A., Luis P., Benea L., Van der Bruggen B., Kim J. (2012). A new outlook on membrane enhancement with nanoparticles: The alternative of ZnO. J. Membr. Sci..

[23] Upadhyaya L., Semsarilar M., Quemener D., Fernández-Pacheco R., Martinez G., Coelhoso I.M., Nunes S.P., Crespo J.G., Mallada R., Portugal C.A.M. (2021). Block Copolymer-Based Magnetic Mixed Matrix Membranes—Effect of Magnetic Field on Protein Permeation and Membrane Fouling. Membranes.

[24] Dimulescu I.A., Nechifor A.C., Bǎrdacǎ C., Oprea O., Paşcu D., Totu E.E., Albu P.C., Nechifor G., Bungău S.G. (2021). Accessible Silver-Iron Oxide Nanoparticles as a Nanomaterial for Supported Liquid Membranes. Nanomaterials.

[25] Huisman I.H., Prádanos P., Hernández A. (2000). The effect of protein–protein and protein–membrane interactions on membrane fouling in ultrafiltration. J. Membr. Sci..

[26] Ghimpusan M., Nechifor G., Nechifor A.C., Dima S.O., Passeri P. (2017). Case studies on the physical-chemical parameters’ variation during three different purification approaches destined to treat wastewaters from food industry. J. Environ. Manag..

[27] Zaman J., Chakma A. (1994). Inorganic membrane reactors. J. Membr. Sci..

[28] Molinari R., Mungari M., Drioli E., Di Paola A., Loddo V., Palmisano L., Schiavello M. (2000). Study on a photocatalytic membrane reactor for water purification. Catal. Today.

[29] Uemiya S., Sato N., Ando H., Matsuda T., Kikuchi E. (1990). Steam reforming of methane in a hydrogen-permeable membrane reactor. Appl. Catal..

[30] Giorno L., Drioli E. (2000). Biocatalytic membrane reactors: Applications and perspectives. Trends Biotechnol..

[31] Molinari R., Pirillo F., Falco M., Loddo V., Palmisano L. (2004). Photocatalytic degradation of dyes by using a membrane reactor. Chem. Eng. Process..

[32] Dubé M.A., Tremblay A.Y., Liu J. (2007). Biodiesel production using a membrane reactor. Bioresour. Technol..

[33] Balachandran U., Dusek J.T., Maiya P.S., Ma B., Mieville R.L., Kleefisch M.S., Udovich C.A. (1997). Ceramic membrane reactor for converting methane to syngas. Catal. Today.

[34] Weyten H., Luyten J., Keizer K., Willems L., Leysen R. (2000). Membrane performance: The key issues for dehydrogenation reactions in a catalytic membrane reactor. Catal. Today.

[35] Wang Y.-K., Sheng G.-P., Li W.-W., Huang Y.-X., Yu Y.-Y., Zeng R.J., Yu H.-Q. (2011). Development of a Novel Bioelectrochemical Membrane Reactor for Wastewater Treatment. Environ. Sci. Technol..

[36] Yin J., Zhan F., Jiao T., Deng H., Zou G., Bai Z., Zhang Q., Peng Q. (2020). Highly efficient catalytic performances of nitro compounds via hierarchical PdNPs-loaded MXene/polymer nanocomposites synthesized through electrospinning strategy for wastewater treatment. Chin. Chem. Lett..

[37] Harish S., Mathiyarasu J., Phani K.L.N., Yegnaraman V. (2009). Synthesis of conducting polymer supported Pd nanoparticles in aqueous medium and catalytic activity towards 4-nitrophenol reduction. Catal. Lett..

[38] Khalil A.M., Georgiadou V., Guerrouache M., Mahouche-Chergui S., Dendrinou-Samara C., Chehimi M.M., Carbonnier B. (2015). Gold- decorated polymeric monoliths: In-situ vs ex-situ immobilization strategies and flow through catalytic applications towards nitrophenols reduction. Polymer.

[39] Kuroda K., Ishida T., Haruta M. (2009). Reduction of 4-nitrophenol to 4-aminophenolover Au nanoparticles deposited on PMMA. J. Mol. Catal. A Chem..

[40] Koga H., Kitaoka T. (2011). One-step synthesis of gold nanocatalysts on a micro-structured paper matrix for the reduction of 4-nitrophenol. Chem. Eng. J..

[41] Dong Z., Le X., Dong C., Zhang W., Li X., Ma J. (2015). Ni@Pd core-shell nanoparticles modified fibrous silica nanospheres as highly efficient and recoverable catalyst for reduction of 4-nitrophenol and hydrodechlorination of 4-chlorophenol. Appl. Catal. B Environ..

[42] Macanás J., Ouyang L., Bruening M.L., Muñoz M., Remigy J.-C., Lahitte J.-F. (2010). Development of polymeric hollow fiber membranes containing catalytic metal nanoparticles. Catal. Today.

[43] Wang H., Dong Z., Na C. (2013). Hierarchical carbon nanotube membrane-supported gold nanoparticles for rapid catalytic reduction of p-nitrophenol. ACS Sustain. Chem. Eng..

[44] Wu W., Liu G., Liang S., Chen Y., Shen L., Zheng H., Yuan R., Hou Y., Wu L. (2012). Efficient visible-light-induced photocatalytic reduction of 4-nitroaniline to p-phenylenediamine over nanocrystalline PbBi_2_Nb_2_O_9_. J. Catal..

[45] Le X., Dong Z., Li X., Zhang W., Le M., Ma J. (2015). Fibrous nano-silica supported palladium nanoparticles: An efficient catalyst for the reduction of 4-nitrophenol and hydrodechlorination of 4-chlorophenol under mild conditions. Catal. Commun..

[46] Fang Y., Wang E. (2013). Simple and direct synthesis of oxygenous carbon supported palladium nanoparticles with high catalytic activity. Nanoscale.

[47] de Pedro Z.M., Diaz E., Mohedano A.F., Casas J.A., Rodriguez J. (2011). Compared activity and stability of Pd/Al2O3 and Pd/AC catalysts in 4-chlorophenol hydrodechlorination in different pH media. J. Appl. Catal. B Environ..

[48] Wang C., Yin J., Han S., Jiao T., Bai Z., Zhou J., Zhang L., Peng Q. (2019). Preparation of Palladium Nanoparticles Decorated Polyethyleneimine/Polycaprolactone Composite Fibers Constructed by Electrospinning with Highly Efficient and Recyclable Catalytic Performances. Catalysts.

[49] Borja-Arco E., Castellanos R.H., Uribe-Godínez J., Altamirano-Gutiérrez A., Jiménez-Sandoval O. (2009). Osmium–ruthenium carbonyl clusters as methanol tolerant electrocatalysts for oxygen reduction. J. Power Sources.

[50] Bolitho E.M., Coverdale J.P.C., Bridgewater H.E., Clarkson G.J., Quinn P.D., Sanchez-Cano C., Sadler P.J. (2021). Tracking Reactions of Asymmetric Organo-Osmium Transfer Hydrogenation Catalysts in Cancer Cells. Angew. Chem. Int. Ed..

[51] Ghimpusan M., Nechifor G., Din I.S., Nechifor A.C., Passeri P. (2016). Application of Hollow Fiber Membrane Bioreactor Instead of Granular Activated Carbon Filtration for Treatment of Wastewater from Car Dismantler Activity. Mat. Plast..

[52] Din I.S., Cimbru A.M., Rikabi A.A.K.K., Tanczos S.K., Ticu S., Nechifor G. (2018). Iono-molecular Separation with Composite Membranes VI. Nitro-phenol separation through sulfonated polyether ether ketone on capillary polypropylene membranes. Rev. Chim..

[53] Batrinescu G., Scutariu R.E., Nechifor G., Ionescu I.A., Iancu V.I. (2021). Comparative analysis of the processes of collagen concentration by ultrafiltration using different types of membranes. J. Appl. Polym. Sci..

[54] Scutariu R.E., Batrinescu G., Nechifor G., Popescu M., Tenea A.G. (2020). Separation of the collagen protein by ultrafiltration: Effects of concentration on the membrane’s characteristics. Polym. Eng. Sci..

[55] Miricioiu M.G., Iacob C., Nechifor G., Niculescu V.C. (2019). High selective mixed membranes based on mesoporous MCM-41 and MCM-41-NH2 particles in a polysulfone matrix. Front. Chem..

[56] Bărdacă Urducea C., Nechifor A.C., Dimulescu I.A., Oprea O., Nechifor G., Totu E.E., Isildak I., Albu P.C., Bungău S.G. (2020). Control of Nanostructured Polysulfone Membrane Preparation by Phase Inversion Method. Nanomaterials.

[57] Grosu A.R., Nafliu I.M., Din I.S., Cimbru A.M., Nechifor G. (2020). Neutralization with simultaneous separation of aluminum and copper ions from condensed water through capillary polypropylene and cellulose. UPB Sci. Bull. Ser. B Chem. Mater. Sci..

[58] Szczepański P., Diaconu I. (2012). Transport of p-nitrophenol through an agitated bulk liquid membrane. Sep. Sci. Technol..

[59] Diaconu I., Nechifor G., Nechifor A.C., Ruse E., Totu E.E. (2009). Membranary techniques used at the separation of some phenolic compounds from aqueous media. Chem. Mater. Sci..

[60] Koter S., Szczepański P., Mateescu M., Nechifor G., Badalau L., Koter I. (2013). Modeling of the cadmium transport through a bulk liquid membrane. Sep. Purif. Technol..

[61] Diaconu I., Gîrdea R., Cristea C., Nechifor G., Ruse E., Totu E.E. (2010). Removal and recovery of some phenolic pollutants using liquid membranes. Rom. Biotechnol. Lett..

[62] Lo Y.M., Cao D., Argin-Soysal S., Wang J., Hahm T.-S. (2005). Recovery of protein from poultry processing wastewater using membrane ultrafiltration. Bioresour. Technol..

[63] Ren J., Li Z., Wong F.-S. (2006). New method for the prediction of pore size distribution and MWCO of ultrafiltration membranes. J. Membr. Sci..

[64] Lowry O., Rosebrough N.J., Farr A.L., Randall R.J. (1951). Protein measurement with the Folin phenol reagent. J. Biol. Chem..

[65] Chelucci G., Baldino S., Baratta W. (2015). Recent Advances in Osmium-Catalyzed Hydrogenation and Dehydrogenation Reactions. Acc. Chem. Res..

[66] Baratta W., Ballico M., Chelucci G., Siega K., Rigo P. (2008). Osmium(II) CNN Pincer Complexes as Efficient Catalysts for Both Asymmetric Transfer and H2 Hydrogenation of Ketones. Angew. Chem. Int. Ed..

[67] George A., Selvan D., Mandal S. (2017). Catalytic Reduction of Toxic Nitroarenes in Aqueous Medium Using Worm-Like Rhodium Nanoparticles. Chem. Sel..

