# Supplementary material for: Reactional Processes on Osmium–Polymeric Membranes for 5–Nitrobenzimidazole Reduction
Source: Membranes (Basel). 2021 Aug 17;11(8):633. doi: 10.3390/membranes11080633 (PMC8400646; doi:10.3390/membranes11080633)
Supplement: Supplementary file 1 [file membranes-11-00633-s001.zip › membranes-1341725-supplementary.pdf]

membranes

Supplementary material

Reactional Processes on Osmium–Polymeric Membranes for 5–Nitrobenzimidazole Reduction

Aurelia Cristina Nechifor [1], Alexandru Goran [1], Vlad-Alexandru Grosu [2,*], Andreia Pîrțac [1], Paul Constantin Albu [3], Ovidiu Oprea [4], Alexandra Raluca Grosu [1,*], Dumitru Pașcu [1], Florentina Mihaela Păncescu [1], Gheorghe Nechifor [1], Szidonia-Katalin Tanczos [5] and Simona Gabriela Bungău [6]

[1] Analytical Chemistry and Environmental Engineering Department, University Politehnica of Bucharest, 1-7 Polizu Street, 011061 Bucharest, Romania; aureliacristinanechifor@gmail.com (A.C.N.); alexandru@santego.ro (A.G.); andreia.pascu@yahoo.ro (A.P.); dd.pascu@yahoo.com (D.P.); florynicorici@yahoo.com (F.M.P.); ghnechifor@gmail.com (G.N.)
[2] Department of Electronic Technology and Reliability, Faculty of Electronics, Telecommunications and Information Technology, University Politehnica of Bucharest, Bulevardul Iuliu Maniu, nr. 1-3, 061071 Bucharest, Romania
[3] IFIN Horia Hulubei, Radioisotopes and Radiation Metrology Department (DRMR), 30 Reactorului Street, 023465 Măgurele, Romania; paulalbu@gmail.com
[4] Department of Inorganic Chemistry, Physical Chemistry and Electrochemistry, University Politehnica of Bucharest, 1-7 Polizu Street, 011061 Bucharest, Romania; ovidiu.oprea@upb.ro
[5] Department of Bioengineering, Sapientia Hungarian University of Transylvania, Libertatii Street, 500104 Miercurea-Ciuc, Romania; tczszidonia@yahoo.com
[6] Department of Pharmacy, Faculty of Medicine and Pharmacy, University of Oradea, 410028 Oradea, Romania; sbungau@uoradea.ro
* Correspondence: vlad.grosu@upb.ro (V.-A.G.); alexandra.raluca.miron@gmail.com (A.R.G.)

Abstract: Membranes are associated with the efficient processes of separation, concentration and purification, but a very important aspect of them is the realization of a reaction process simultaneously with the separation process. From a practical point of view, chemical reactions have been introduced in most membrane systems: with on liquid membranes, with inorganic membranes or with polymeric and/or composite membranes. This paper presents the obtaining of polymeric membranes containing metallic osmium obtained in situ. Cellulose acetate (CA), polysulfone (PSf) and polypropylene hollow fiber membranes (PPM) were used as support polymer membranes. The metallic osmium is obtained directly onto the considered membranes using a solution of osmium tetroxide (OsO4), dissolved in tert–butyl alcohol (t–Bu–OH) by reduction with molecular hydrogen. The composite osmium–polymer (Os–P) obtained membranes were characterized in terms of the morphological and structural points of view: scanning electron microscopy (SEM), high resolution SEM (HR–SEM), energy dispersive spectroscopy analysis (EDAX), Fourier Transform Infra-Red (FTIR) spectroscopy, thermogravimetric analysis (TGA) and differential scanning calorimetry (DSC). The process performance was tested were tested for reduction of 5–nitrobenzimidazole to 5–aminobenzimidazole with molecular hydrogen. The paper presents the main aspects of the possible mechanism of transformation of 5–nitrobenzimidazole to 5–aminobenzimidazole with hydrogen gas in the reaction system with osmium–polymer membrane (Os–P).

Keywords: composite membranes; osmium polymer membrane; nitro derivatives reduction; reactional processes; 5–nitrobenzimidazole; cellulose acetate membranes; polysulfone membranes; polypropylene hollow fiber membranes.

Scanning electron microscopy (SEM), high resolution SEM (HR–SEM) for the osmium–polymer membranes

The microscopy studies, SEM and HFSEM, were performed on a Hitachi S4500 system (Hitachi High-Technologies Europe GmbH, Krefeld, Germany).

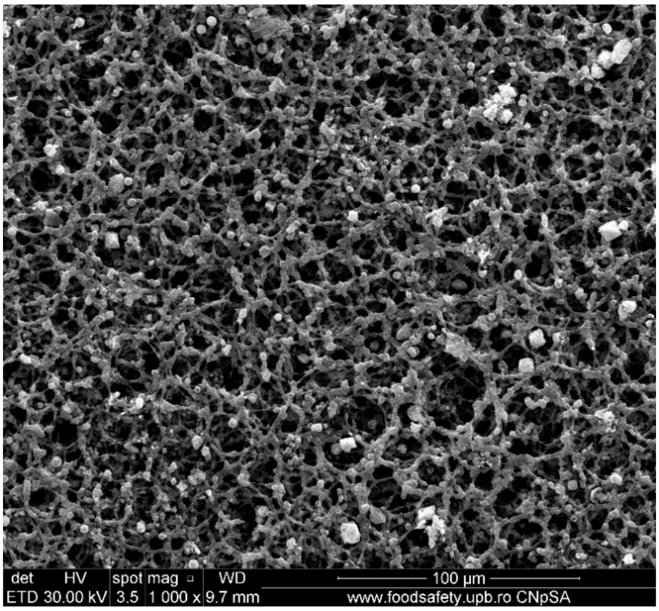
det HV spot mag WD
ETD 30.00 kV 3.5 1 000 x 9.7 mm
100 µm
www.foodsafety.upb.ro CNpSA

(a)

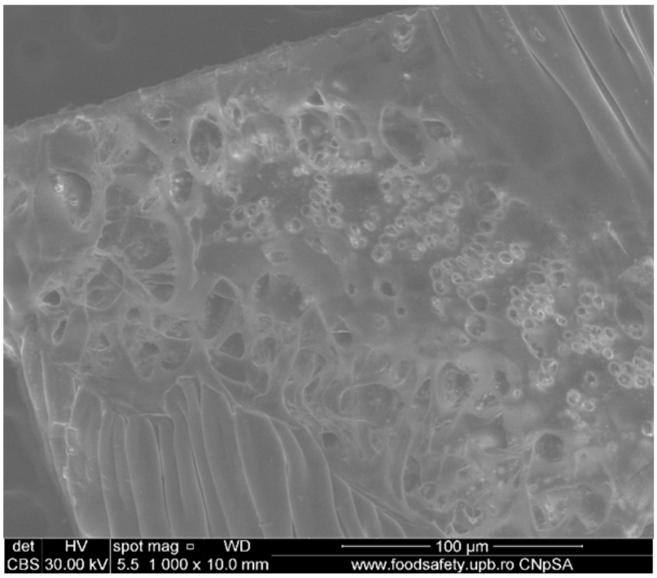
det HV spot mag WD
CBS 30.00 kV 5.5 1 000 x 10.0 mm
100 µm
www.foodsafety.upb.ro CNpSA

(b)

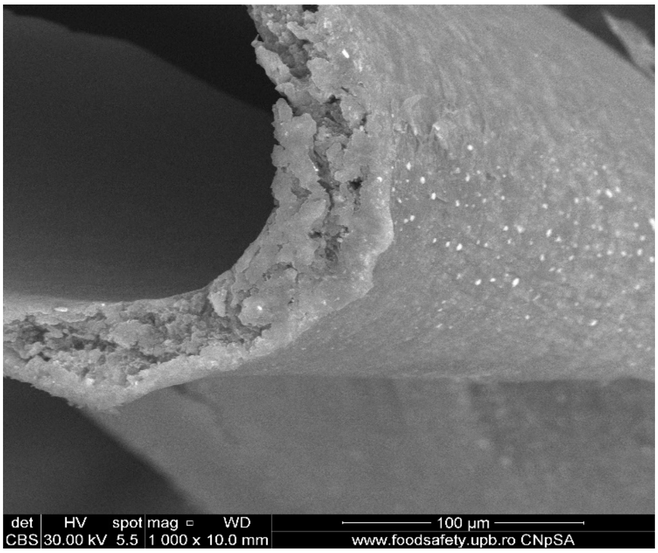
det HV spot mag WD
CBS 30.00 kV 5.5 1 000 x 10.0 mm
100 µm
www.foodsafety.upb.ro CNpSA

(c)

Figure S1. The scanning electron microscopy on the cross-section of the osmium–polymer membrane (Os–P): **(a)** osmium-cellulose acetate (Os–CA); **(b)** osmium-polysulfone (Os–PSf) and **(c)** osmium-polypropylene membranes (Os–PP).

Thermal analysis for the osmium–polymer membranes

Thermal characterizations were performed on a Netzsch Thermal Analyzer (Netzsch – Gerätebau GmbH, Selb, Germany). The thermal analysis was run in a nitrogen atmosphere at 10 °C/min heating rate, from the room temperature (RT=25 °C) up to 900 °C (Figures S2 to S4).

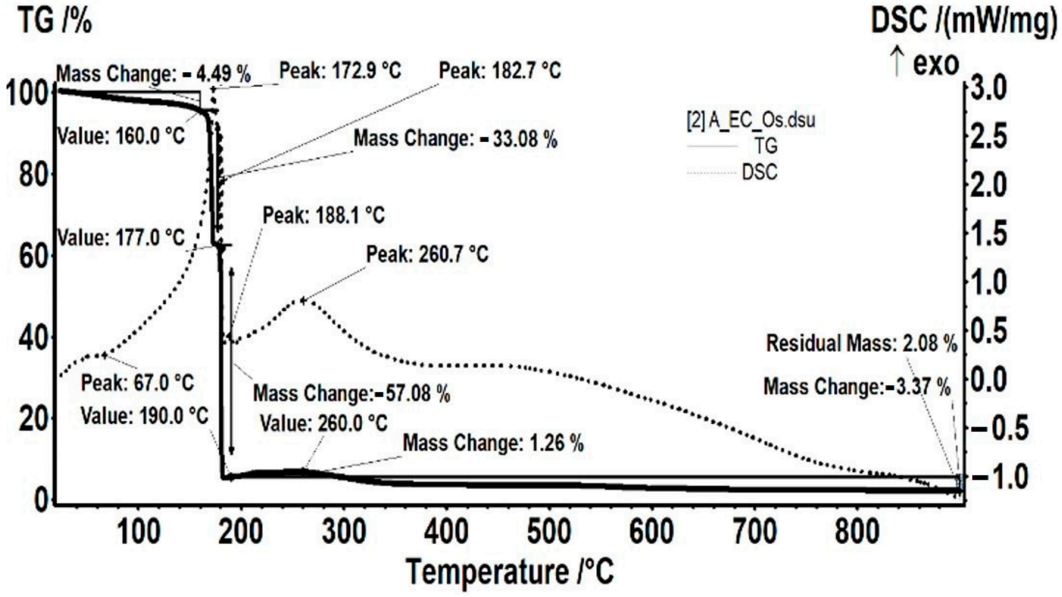

(a)

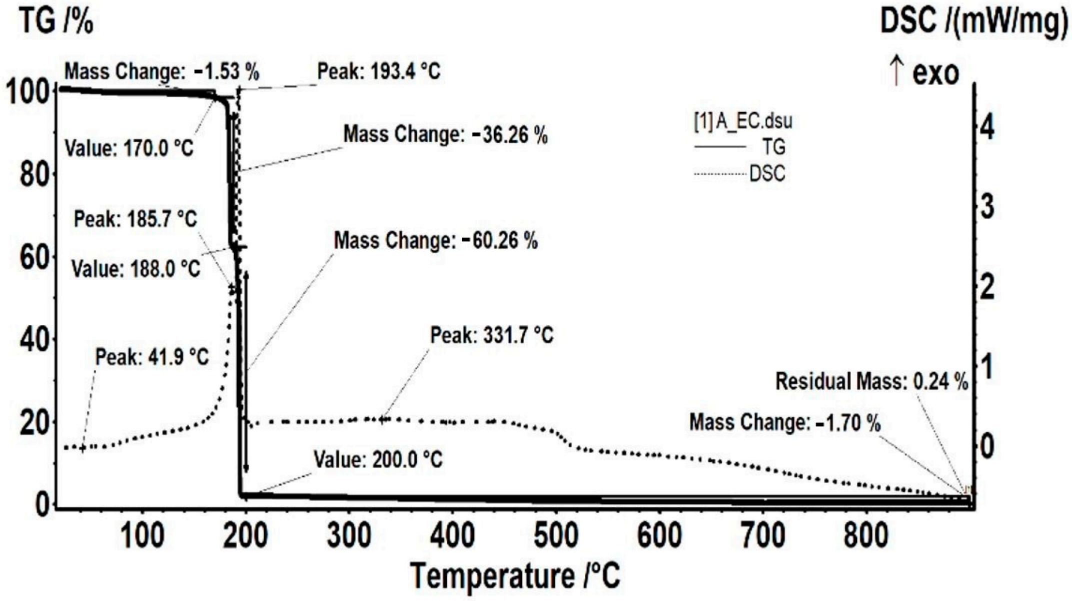

(b)

Figure S2. The thermal diagrams for: osmium-cellulose acetate membrane (Os–CA) **(a)**; and cellulose acetate (CA) **(b)**.

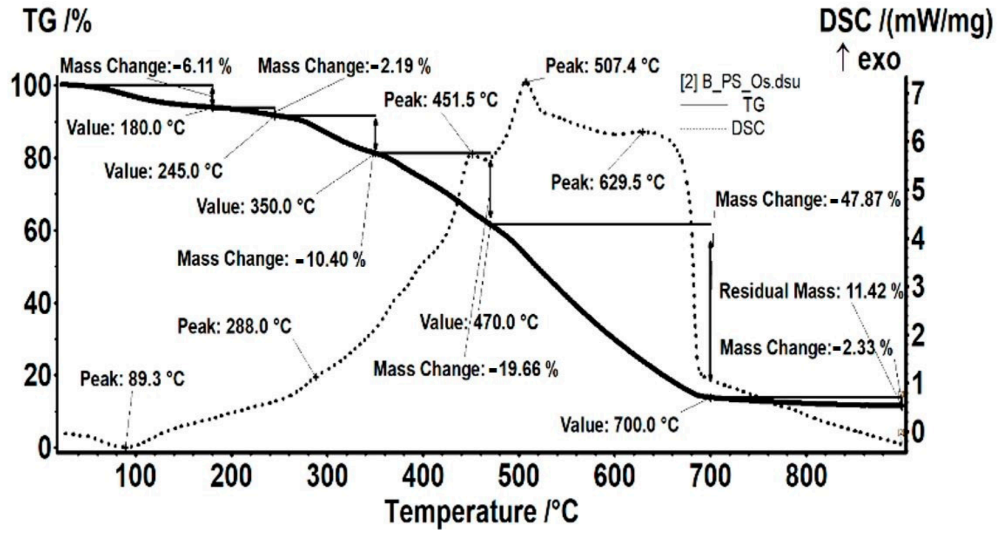

(a)

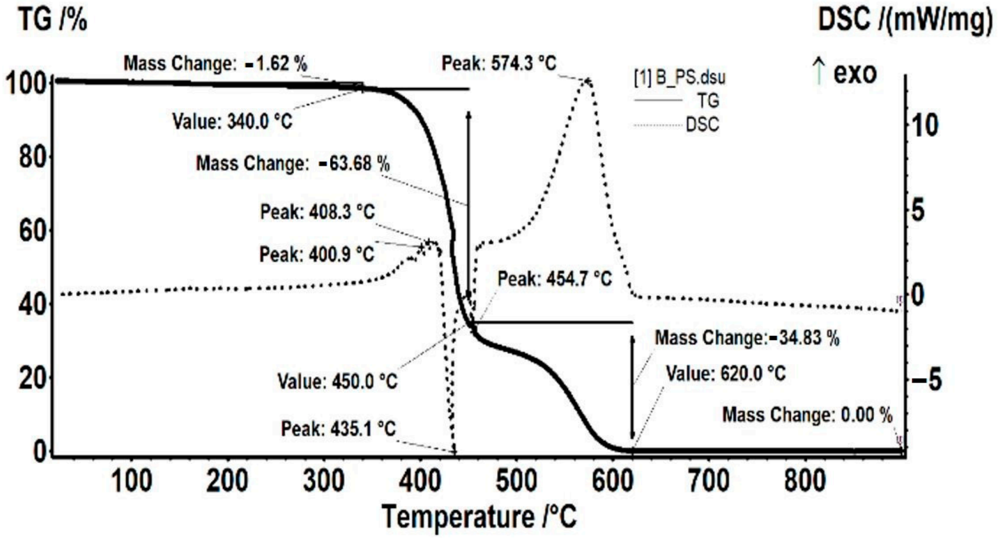

(b)

Figure S3. The thermal diagrams for: osmium-polysulfone membrane (Os–PSf) **(a)**; and polysulfone (PSF) **(b)**.

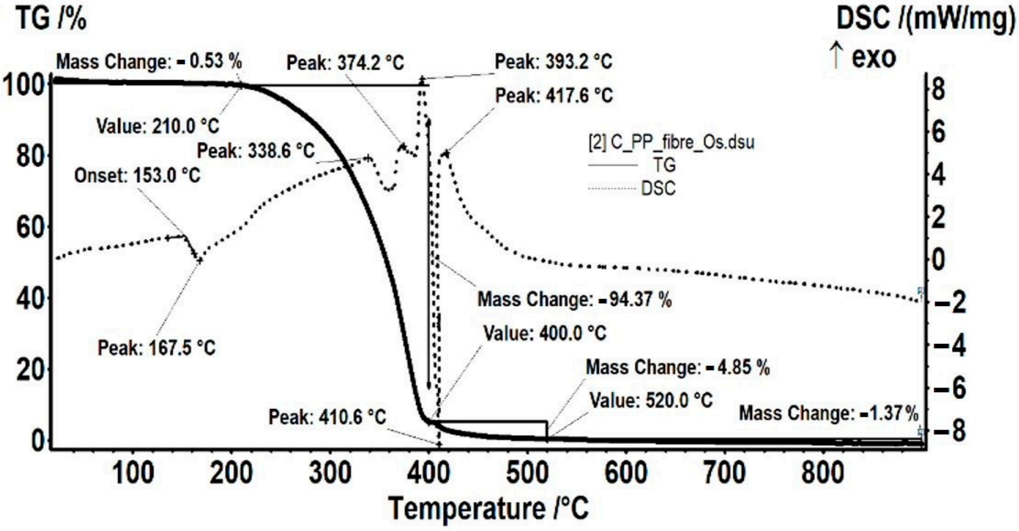

(a)

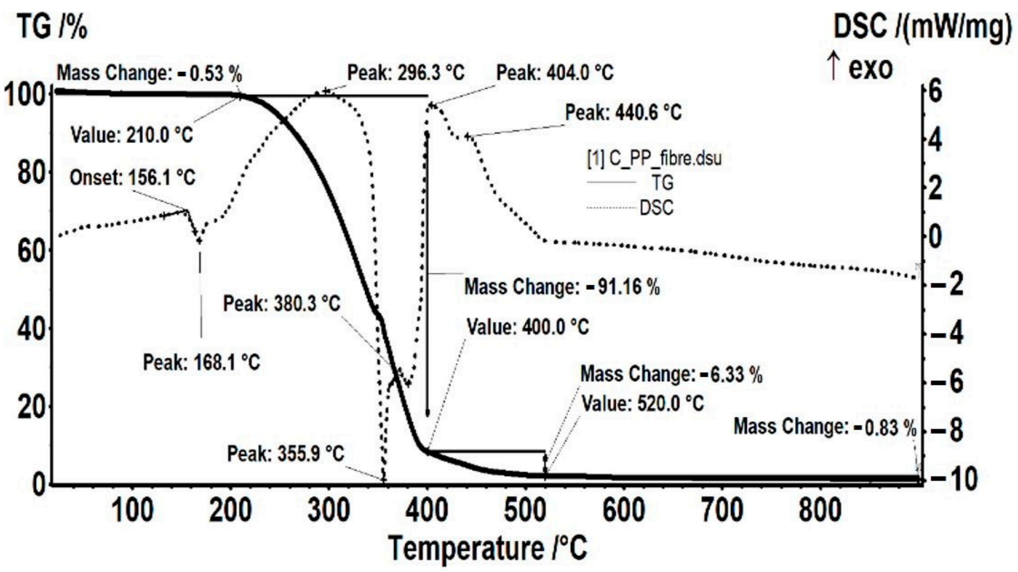

(b)

Figure S4. The thermal diagrams for: osmium-polypropylene membrane (Os–PP) **(a)**; and hollow fiber polypropylene membrane (PP) **(b)**.

3. Compositional characterization of membranes

Energy dispersive spectroscopy analysis (EDAX) for the osmium–polymer membranes provide the surface distribution of osmium; its appearance being shown in Figure S5.

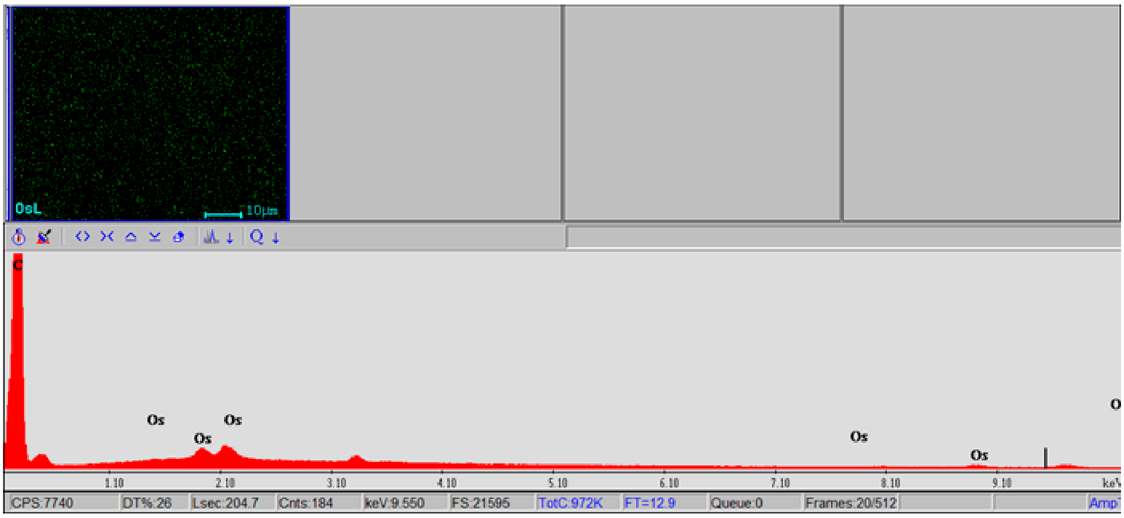

(a)

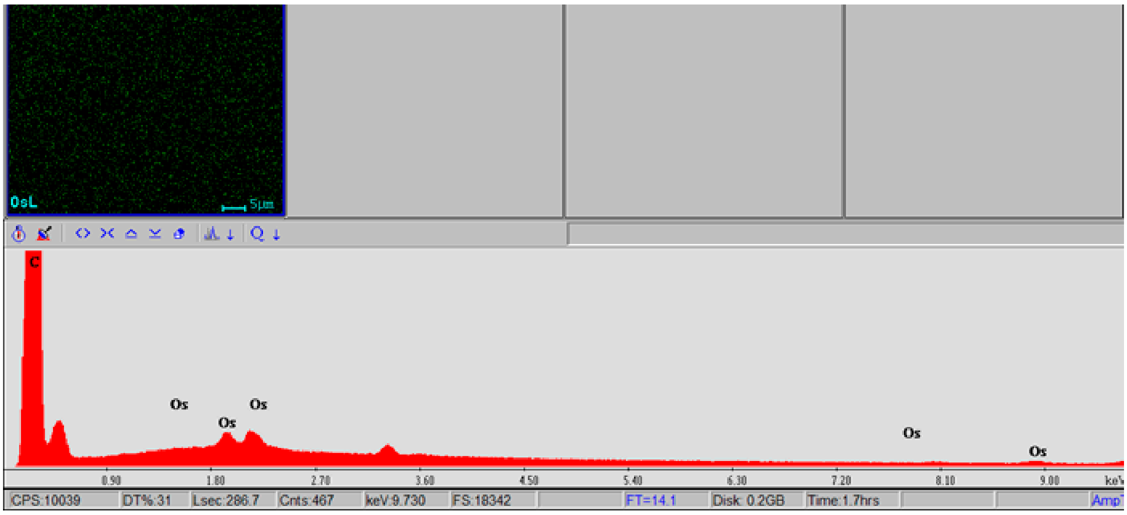

(b)

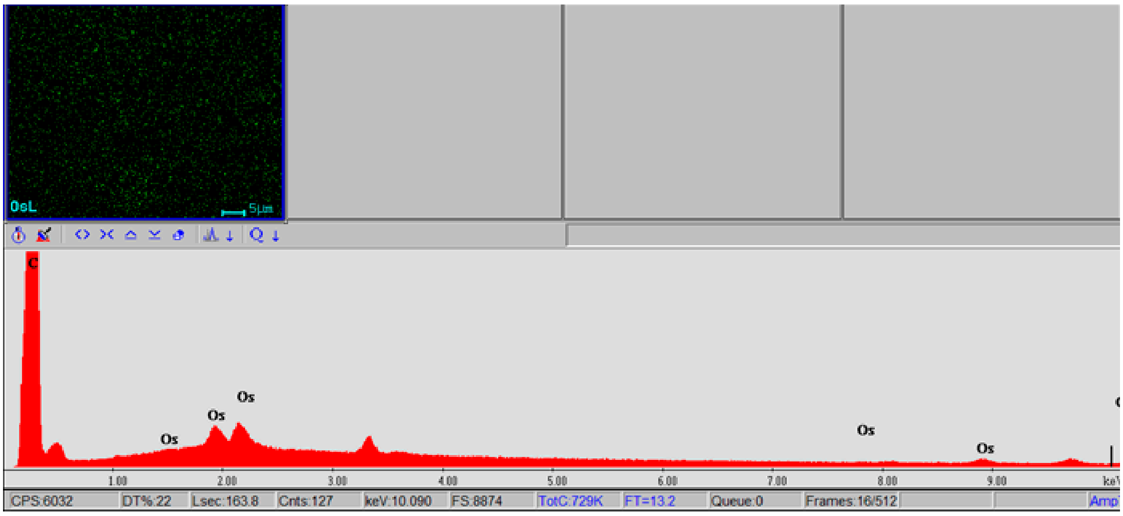

(c)

Figure S5. Formal energy dispersive spectroscopy analysis (EDAX) for the osmium distribution on the osmium–polymer membranes: **(a)** osmium–cellulose acetate (Os–CA); **(b)** osmium–polysulfone (Os–PSf); and **(c)** osmium–polypropylene membranes (Os–PP).

Funding: This research received no external funding.

Institutional Review Board Statement: Not applicable.

Informed Consent Statement: Not applicable.

Data Availability Statement: Not applicable.

Acknowledgments: The authors gratefully acknowledge the valuable help and friendly assistance of Eng. Roxana Truşcă for performing the scanning microscopy analysis.

Conflicts of Interest: The authors declare no conflict of interest.
